# Exploration of adverse event profiles for glofitamab: A disproportionality analysis using the FDA adverse event reporting system

**DOI:** 10.1371/journal.pone.0336151

**Published:** 2025-11-04

**Authors:** Meng Zhou, Cheng Jiang, Chuanyong Su

**Affiliations:** 1 Zhejiang Academy of Traditional Chinese Medicine, Tongde Hospital of Zhejiang Province, Hangzhou, Zhejiang, China; 2 Zhejiang Provincial Key Laboratory of Disease-Syndrome Integrated Cancer Prevention and Treatment, Tongde Hospital of Zhejiang Province, Hangzhou, Zhejiang, China; 3 Zhejiang Provincial Key Laboratory of Traditional Chinese Medicine for Pharmacodynamic Material Basis Research of Chinese Medicine, Tongde Hospital of Zhejiang Province, Hangzhou, Zhejiang, China; University of California Santa Barbara, UNITED STATES OF AMERICA

## Abstract

**Background:**

Glofitamab offers a promising option for the treatment of diffuse large B-cell lymphoma. It is crucial to gather comprehensive safety information of glofitamab through large-scale post market monitoring.

**Methods:**

This study conducted a comprehensive analysis of glofitamab-related adverse events (AEs) based on FDA Adverse Event Reporting System database. Four disproportionality analysis methods were employed to mining the significant signals. The clinical characteristics of all AE and cytokine release syndrome reports were analyzed. Sensitivity analyses were performed to exam the potential bias. The differences in AE signals among different subgroups were investigated.

**Results:**

A total of 641 reports and 1,542 AEs with glofitamab were identified. Cytokine release syndrome was the most significant signal. Notably, American and European reporters demonstrated higher cytokine release syndrome reporting frequency. Cytokine release syndrome was often reported by professionals and occurred within 30 days, especially with glofitamab at a dose of 2.5 mg. Hypogammaglobulinaemia was discovered as a new significant AE signal.

**Conclusions:**

The findings suggest potential reporting differences in glofitamab-related AEs across different continents. Educating consumers on how to recognize the early symptoms of cytokine release syndrome is essential to improve safety. Close monitoring of cytokine release syndrome is recommended within 30 days of administration of glofitamab, especially at a dose of 2.5 mg. Furthermore, it is essential to stay vigilant about the emergence of the newly identified AE. These findings contribute to a broader understanding of the AE profiles of glofitamab.

## Introduction

Diffuse large B-cell lymphoma (DLBCL) is the most prevalent subtype of non-Hodgkin lymphoma (NHL), representing about 30% of all NHL cases globally [1]. According to data from the United States cancer registry, the age-standardized incidence rate of DLBCL is 7.2 per 100,000 individuals [2]. Current estimates indicate that nearly 150,000 new cases of DLBCL are diagnosed each year worldwide, placing a substantial burden on healthcare systems around the globe [3]. The pathogenesis of DLBCL is complex and multifactorial, involving genetic alterations, epigenetic modifications, and dysregulated signaling pathways [4].

Present treatment options primarily consist of chemotherapy regimens like CHOP (cyclophosphamide, doxorubicin, vincristine, and prednisone) combined with rituximab, an anti-CD20 monoclonal antibody, have boosted general survival rates [5]. Nevertheless, a large number of patients still suffer from recurrence or treatment-resistant disease [3,6], highlighting the urgent need for new therapeutic strategies. Integrating biomarker-driven approaches and targeted therapies, such as bispecific antibodies, has proven to be a promising way of enhancing treatment efficacy and managing the adverse effects associated with conventional therapies.

Glofitamab is a novel bispecific antibody that targets both CD20 and CD3, representing a significant advancement in the treatment of relapsed or refractory DLBCL. This innovative therapy works by engaging T-cells to eliminate malignant B-cells, effectively disrupting critical pathways involved in the pathology of DLBCL. By inhibiting these pathways, glofitamab not only promotes effective anti-tumor responses but also offers a new strategic approach to treat patients with DLBCL, potentially improving their overall survival [7]. The United States Food and Drug Authority approved glofitamab for treating relapsed or refractory DLBCL on June 15, 2023, marking a pivotal moment in the therapeutic landscape for this challenging condition [7].

Glofitamab has been widely used in clinical practice, with numerous reports documenting its application across various patients [8,9]. However, there has been a gradual increase in reports of related adverse events (AEs) [10]. Despite these concerns, it is important to note that the majority of safety data on glofitamab originates from short-term clinical trials, case reports or meta-analyses [11–15]. Due to the strict diagnostic and selection criteria, AEs are often concentrated on a single or multiple systems, and the relatively small sample sizes limit the observable AEs. Additionally, the long-term use of glofitamab may lead to new or serious safety concerns, and the time to onset of AEs often remains unknown [9]. Therefore, it is essential to explore the potential AE signals of glofitamab through data mining algorithms using large-sample post-marketing monitoring, highlighting the significance and necessity of this investigation.

The FDA Adverse Event Reporting System (FAERS) is among the largest databases globally for monitoring AEs related to drugs, encompassing tens of millions of case reports submitted by a variety of sources, including patients, pharmaceutical companies, physicians, and pharmacists [16]. The present study aimed to evaluate the AEs associated with glofitamab since its approval in the market, utilizing the FAERS database to enhance the understanding of glofitamab’s safety profile.

## Materials and methods

### Data source and collection

This AE profile study of glofitamab spans from the second quarter of 2023 (the quarter in which glofitamab received FDA approval) to the first quarter of 2025 (the most recent quarter available at the time of study initiation, which was posted on April 28, 2025). Reports were included if glofitamab was identified as the primary suspect (PS) drug, determined by searching for “GLOFITAMAB” or “GLOFITAMABB-GXBM” in the “prod_ai” column and “PS” in the “role_cod” column. The preferred terms (PTs) for glofitamab-associated AEs were mapped to their corresponding primary System Organ Class (SOC) categories based on the standardized Medical Dictionary for Regulatory Activities (MedDRA), version 27.0. Additional details regarding data sources and collection methods are available in our previously published articles [17–21].

### Statistical analysis

The AE signals associated with glofitamab were detected if it simultaneously met the detection criteria across four algorithms: reporting odds ratio (ROR) [22,23], proportional reporting ratio (PRR) [24,25], Bayesian confidence propagation neural network (BCPNN) [24–26], and the multi-item gamma Poisson shrinker (MGPS) [24–26]. Descriptive analyses were employed to thoroughly examine the clinical characteristics of both all AE and cytokine release syndrome (CRS) reports after removing missing data.

To evaluate the robustness of these signals against potential bias, sensitivity analyses were performed. Firstly, only glofitamab-associated AE reports without concomitant medication were included to eliminate the influence of co-administered drugs. Secondly, the glofitamab-associated AE reports with diffuse large B-cell lymphoma as the treatment indication were analyzed to control for the effects of unrelated underlying conditions. Thirdly, considering the reliability of reports, a stratified analysis was conducted based solely on reports submitted by professionals due to their clinical expertise. Fourthly, additional stratified analyses were performed by sex and age, to explore potential demographic differences. Fifthly, given the known reporting biases in the FAERS database (such as comprehensive AE reporting from the United States and serious AE reporting from other countries), continent-based stratified analyses were conducted to minimize geographic reporting bias.

Based on the main findings from signal detection and clinical characterization, subgroup analyses were conducted to explore differences in glofitamab-associated AE signals across diverse clinical characteristics. Significant signal difference was identified when a signal met both the criteria of ROR and the Chi-square test or Fisher’s exact test. The fourfold tables, statistical equations and signal detection criteria are detailed in S1–S4 Tables and our previously published articles [17–21]. All data processing and statistical analyses were performed using Python 3 programming language in Jupyter Notebook, version 6.4.12.

## Results

### Signals detection

**Fig 1:**
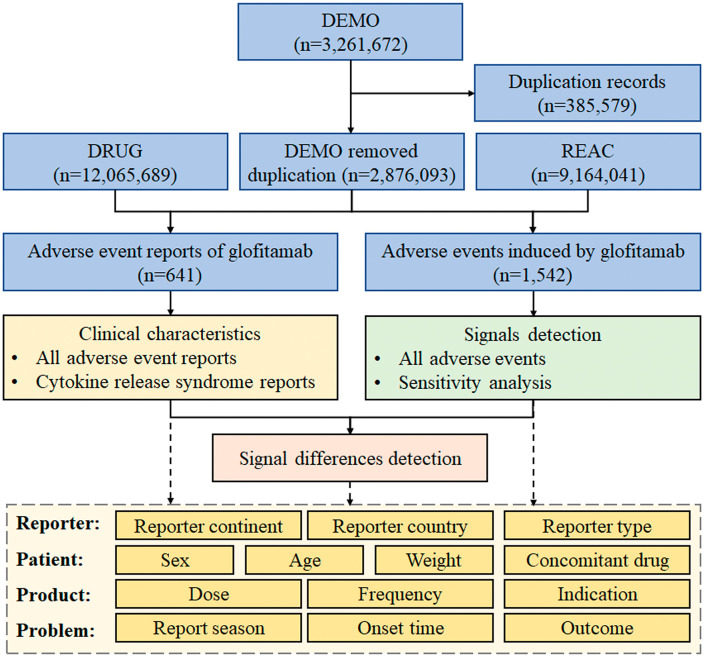
Flow diagram of data collection and analysis of glofitamab-associated adverse events. Abbreviations: DEMO, patient demographic and administrative information; DRUG, drug information; REAC, coded for the adverse events.

A total of 641 reports and 1,542 AEs induced by glofitamab were identified. A flow diagram of data collection and analysis of glofitamab-associated AEs is shown in **Fig 1**.

The number and signal strength of glofitamab at the SOC level are described in Table 1. Glofitamab-associated AEs involved 24 SOCs. Statistically, the most significant SOC that met four criteria was “Immune system disorders” [n: 168, ROR (95% CI): 10.00 (8.52–11.74), PRR (χ2): 9.02 (1210.38), IC (IC025): 3.17 (2.87), EBGM (EBGM05): 9.01 (7.67)]. A total of 48 signals at the PT level conformed to the four algorithms simultaneously. Among these, there were 8 signals of “Neoplasms benign, malignant and unspecified (incl cysts and polyps)”, 1 signal of “Disease progression”, and 1 signal of “No adverse event”, which might be caused by original disease, disease progression or other factors. The number and signal strength of 10 glofitamab-unrelated signals at the PT level are listed in S5 Table. After excluding the 10 glofitamab-unrelated signals, 38 glofitamab-related signals at the PT level are shown in Table 2. CRS emerged as the most significant signal associated with glofitamab [n: 154, ROR (95% CI): 150.90 (127.54–178.53), PRR (χ2): 135.93 (20179.79), IC (IC025): 7.05 (5.92), EBGM (EBGM05): 132.91 (112.34)]. Notably, novel glofitamab-related signals uncovered in the label and clinical trials were highlighted, namely “hypogammaglobulinaemia”.

**Table 1:** Number and signal strength of glofitamab at the SOC level.

SOC	Number	ROR (95% CI)	PRR (χ2)	IC (IC025)	EBGM (EBGM05)
General disorders and administration site conditions (SOC: 10018065) ^ac^	374	1.51 (1.34-1.69)	1.38 (48.42)	0.47 (0.30)	1.38 (1.23)
Infections and infestations (SOC: 10021881) ^abc^	201	2.29 (1.98-2.66)	2.13 (127.68)	1.09 (0.86)	2.13 (1.83)
Immune system disorders (SOC: 10021428) ^abcd^	168	10.00 (8.52-11.74)	9.02 (1210.38)	3.17 (2.87)	9.01 (7.67)
Investigations (SOC: 10022891) ^ac^	148	1.72 (1.45-2.04)	1.65 (40.29)	0.72 (0.47)	1.65 (1.39)
Blood and lymphatic system disorders (SOC: 10005329) ^abcd^	93	3.61 (2.93-4.45)	3.45 (164.88)	1.79 (1.44)	3.45 (2.80)
Neoplasms benign, malignant and unspecified (incl cysts and polyps) (SOC: 10029104) ^abcd^	87	3.27 (2.63-4.06)	3.14 (129.20)	1.65 (1.30)	3.14 (2.53)
Nervous system disorders (SOC: 10029205)	82	0.73 (0.58-0.91)	0.74 (7.80)	−0.43 (−0.75)	0.74 (0.60)
Injury, poisoning and procedural complications (SOC: 10022117)	79	0.33 (0.27-0.42)	0.37 (100.49)	−1.45 (−1.77)	0.37 (0.29)
Respiratory, thoracic and mediastinal disorders (SOC: 10038738)	66	0.92 (0.72-1.18)	0.92 (0.45)	−0.12 (−0.48)	0.92 (0.72)
Gastrointestinal disorders (SOC: 10017947)	52	0.39 (0.30-0.52)	0.41 (46.75)	−1.27 (−1.66)	0.41 (0.31)
Metabolism and nutrition disorders (SOC: 10027433) ^ac^	47	1.55 (1.16-2.07)	1.53 (8.94)	0.62 (0.18)	1.53 (1.15)
Vascular disorders (SOC: 10047065)	27	0.96 (0.66-1.41)	0.96 (0.04)	−0.06 (−0.61)	0.96 (0.66)
Cardiac disorders (SOC: 10007541)	24	0.84 (0.56-1.25)	0.84 (0.76)	−0.25 (−0.83)	0.84 (0.56)
Renal and urinary disorders (SOC: 10038359)	22	0.92 (0.60-1.40)	0.92 (0.15)	−0.12 (−0.72)	0.92 (0.61)
Hepatobiliary disorders (SOC: 10019805)	17	1.21 (0.75-1.96)	1.21 (0.64)	0.28 (−0.42)	1.21 (0.75)
Musculoskeletal and connective tissue disorders (SOC: 10028395)	15	0.18 (0.11-0.30)	0.19 (54.49)	−2.39 (−3.05)	0.19 (0.11)
Psychiatric disorders (SOC: 10037175)	14	0.18 (0.11-0.31)	0.19 (51.05)	−2.40 (−3.07)	0.19 (0.11)
Skin and subcutaneous tissue disorders (SOC: 10040785)	14	0.16 (0.09-0.26)	0.16 (63.66)	−2.62 (−3.28)	0.16 (0.10)
Eye disorders (SOC: 10015919)	4	0.12 (0.05-0.32)	0.12 (25.28)	−3.01 (−4.03)	0.12 (0.05)
Surgical and medical procedures (SOC: 10042613)	2	0.07 (0.02-0.30)	0.08 (22.85)	−3.72 (−4.85)	0.08 (0.02)
Social circumstances (SOC: 10041244)	2	0.26 (0.06-1.03)	0.26 (4.28)	−1.95 (−3.21)	0.26 (0.06)
Ear and labyrinth disorders (SOC: 10013993)	2	0.31 (0.08-1.23)	0.31 (3.13)	−1.70 (−2.99)	0.31 (0.08)
Reproductive system and breast disorders (SOC: 10038604)	1	0.11 (0.02-0.78)	0.11 (7.21)	−3.18 (−4.37)	0.11 (0.02)
Product issues (SOC: 10077536)	1	0.03 (0.00-0.21)	0.03 (31.44)	−5.03 (−6.12)	0.03 (0.00)

^a^: SOCs met the criteria of ROR algorithm. ^b^: SOCs met the criteria of PRR algorithm. ^c^: SOCs met the criteria of BCPNN algorithm. ^d^: SOCs met the criteria of MGPS algorithm. **Abbreviations:** SOC, system organ class; ROR, reporting odds ratio; CI, confidence interval; PRR, proportional reporting ratio; χ2, chi-squared; IC, information component; IC025, lower limit of 95% confidence interval of IC; EBGM, empirical Bayesian geometric mean; EBGM05, lower limit of 95% confidence interval of EBGM.

**Table 2:** Number and signal strength of 38 glofitamab-related signals at the PT level.

PT	Number	ROR (95% CI)	PRR (χ2)	IC (IC025)	EBGM (EBGM05)
**Immune system disorders (SOC: 10021428)**					
Cytokine release syndrome (PT: 10052015)	154	150.90 (127.54-178.53)	135.93 (20179.79)	7.05 (5.92)	132.91 (112.34)
Hypogammaglobulinaemia (PT: 10020983)*	6	23.42 (10.49-52.29)	23.33 (127.77)	4.54 (1.38)	23.24 (10.41)
Haemophagocytic lymphohistiocytosis (PT: 10071583)	4	10.35 (3.88-27.65)	10.33 (33.65)	3.37 (0.55)	10.31 (3.86)
**General disorders and administration site conditions (SOC: 10018065)**					
Death (PT: 10011906)	78	4.07 (3.24-5.11)	3.91 (171.05)	1.97 (1.58)	3.91 (3.11)
Pyrexia (PT: 10037660)	63	7.98 (6.20-10.27)	7.69 (368.23)	2.94 (2.43)	7.68 (5.97)
Hyperpyrexia (PT: 10020741)	9	73.88 (38.22-142.84)	73.46 (635.44)	6.18 (2.23)	72.57 (37.54)
Multiple organ dysfunction syndrome (PT: 10077361)	6	6.49 (2.91-14.48)	6.47 (27.74)	2.69 (0.77)	6.46 (2.90)
Organ failure (PT: 10053159)	4	61.89 (23.08-165.96)	61.73 (236.56)	5.93 (0.93)	61.11 (22.79)
Temperature intolerance (PT: 10057040)	3	10.25 (3.30-31.83)	10.23 (24.94)	3.35 (0.18)	10.21 (3.29)
**Infections and infestations (SOC: 10021881)**					
Pneumonia (PT: 10035664)	25	3.03 (2.04-4.50)	3.00 (33.48)	1.58 (0.91)	3.00 (2.02)
Infection (PT: 10021789)	22	5.39 (3.54-8.21)	5.32 (77.41)	2.41 (1.56)	5.32 (3.49)
Septic shock (PT: 10040070)	13	11.86 (6.87-20.48)	11.77 (127.93)	3.55 (1.96)	11.75 (6.80)
Cytomegalovirus infection reactivation (PT: 10058666)	4	15.00 (5.62-40.08)	14.97 (52.01)	3.90 (0.68)	14.93 (5.59)
COVID-19 pneumonia (PT: 10084380)	4	7.62 (2.85-20.34)	7.60 (22.92)	2.93 (0.42)	7.59 (2.85)
Pneumocystis jirovecii pneumonia (PT: 10073755)	3	8.66 (2.79-26.90)	8.64 (20.26)	3.11 (0.12)	8.63 (2.78)
Urosepsis (PT: 10048709)	3	14.25 (4.59-44.30)	14.23 (36.81)	3.83 (0.28)	14.19 (4.57)
Cytomegalovirus infection (PT: 10011831)	3	6.83 (2.20-21.20)	6.81 (14.87)	2.77 (0.03)	6.81 (2.19)
Pneumonia fungal (PT: 10061354)	3	21.29 (6.84-66.21)	21.25 (57.68)	4.40 (0.36)	21.17 (6.81)
Disseminated tuberculosis (PT: 10013453)	3	53.15 (17.04-165.83)	53.05 (151.87)	5.72 (0.47)	52.59 (16.86)
**Investigations (SOC: 10022891)**					
Platelet count decreased (PT: 10035528)	19	7.01 (4.46-11.03)	6.94 (96.65)	2.79 (1.77)	6.93 (4.41)
Alanine aminotransferase increased (PT: 10001551)	13	11.35 (6.57-19.60)	11.26 (121.38)	3.49 (1.92)	11.24 (6.51)
White blood cell count decreased (PT: 10047942)	11	3.93 (2.17-7.11)	3.91 (23.85)	1.97 (0.82)	3.91 (2.16)
Aspartate aminotransferase increased (PT: 10003481)	9	9.18 (4.76-17.68)	9.13 (65.10)	3.19 (1.41)	9.12 (4.73)
Blood lactate dehydrogenase increased (PT: 10005630)	7	25.80 (12.26-54.30)	25.69 (165.42)	4.68 (1.63)	25.58 (12.16)
Blood bilirubin increased (PT: 10005364)	7	14.63 (6.96-30.77)	14.57 (88.27)	3.86 (1.41)	14.54 (6.91)
Neutrophil count decreased (PT: 10029366)	6	4.81 (2.16-10.73)	4.80 (18.04)	2.26 (0.54)	4.79 (2.15)
SARS-CoV-2 test positive (PT: 10084271)	4	6.87 (2.57-18.34)	6.86 (19.99)	2.78 (0.36)	6.85 (2.57)
**Blood and lymphatic system disorders (SOC: 10005329)**					
Neutropenia (PT: 10029354)	29	7.14 (4.94-10.31)	7.02 (149.98)	2.81 (2.01)	7.01 (4.86)
Anaemia (PT: 10002034)	19	4.76 (3.03-7.49)	4.72 (55.77)	2.24 (1.34)	4.72 (3.00)
Thrombocytopenia (PT: 10043554)	16	5.99 (3.66-9.81)	5.94 (65.82)	2.57 (1.50)	5.94 (3.63)
Leukocytosis (PT: 10024378)	3	7.99 (2.57-24.83)	7.98 (18.29)	2.99 (0.09)	7.97 (2.57)
**Nervous system disorders (SOC: 10029205)**					
Immune effector cell-associated neurotoxicity syndrome (PT: 10083347)	24	59.11 (39.42-88.64)	58.21 (1336.65)	5.85 (3.56)	57.65 (38.45)
Neurotoxicity (PT: 10029350)	7	14.88 (7.08-31.30)	14.82 (90.00)	3.89 (1.42)	14.78 (7.03)
Movement disorder (PT: 10028035)	7	9.92 (4.72-20.86)	9.88 (55.80)	3.30 (1.20)	9.86 (4.69)
Cerebral haemorrhage (PT: 10008111)	4	6.29 (2.36-16.80)	6.28 (17.74)	2.65 (0.32)	6.27 (2.35)
**Respiratory, thoracic and mediastinal disorders (SOC: 10038738)**					
Hypoxia (PT: 10021143)	6	7.25 (3.25-16.17)	7.23 (32.16)	2.85 (0.84)	7.22 (3.24)
Pneumonitis (PT: 10035742)	5	6.24 (2.59-15.03)	6.23 (21.93)	2.64 (0.55)	6.22 (2.58)
Tachypnoea (PT: 10043089)	3	9.03 (2.91-28.06)	9.02 (21.35)	3.17 (0.14)	9.00 (2.90)
**Metabolism and nutrition disorders (SOC: 10027433)**					
Tumour lysis syndrome (PT: 10045170)	8	31.91 (15.90-64.05)	31.75 (237.06)	4.98 (1.88)	31.59 (15.74)
Hypophosphataemia (PT: 10021058)	5	24.73 (10.26-59.61)	24.66 (113.03)	4.62 (1.13)	24.56 (10.19)
**Hepatobiliary disorders (SOC: 10019805)**					
Hypertransaminasaemia (PT: 10068237)	4	11.38 (4.26-30.40)	11.36 (37.71)	3.50 (0.59)	11.34 (4.25)

*: Novel glofitamab-related signals. **Abbreviations:** PT, preferred term; ROR, reporting odds ratio; CI, confidence interval; PRR, proportional reporting ratio; χ2, chi-squared; IC, information component; IC025, lower limit of 95% confidence interval of IC; EBGM, empirical Bayesian geometric mean; EBGM05, lower limit of 95% confidence interval of EBGM.

### Clinical characteristics regarding reporter

**Fig 2:**
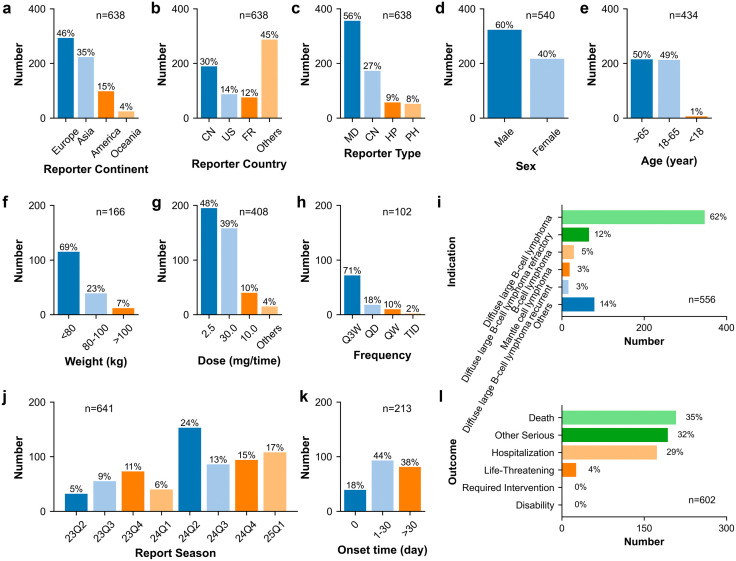
Clinical characteristics of glofitamab-associated adverse event reports. **(a)** Reporter continent. **(b)** Reporter country. **(c)** Reporter type. **(d)** Sex. **(e)** Age. **(f)** Weight. **(g)** Dose. **(h)** Frequency. **(i)** Indication. **(j)** Report season. **(k)** Onset time. **(l)** Outcome. Abbreviations: CN, China; US, United States; FR: France; MD, Physician; CN, consumer; HP, Health-professional; PH, pharmacist; Q3W, once every 3 weeks; QD, once a day; QW, once a week; TID, three times a day.

**Fig 3:**
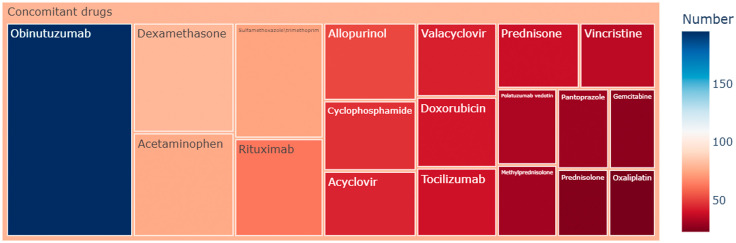
Rectangular tree plot of top 20 ranked concomitant drugs of glofitamab-associated adverse event reports.

**Fig 4:**
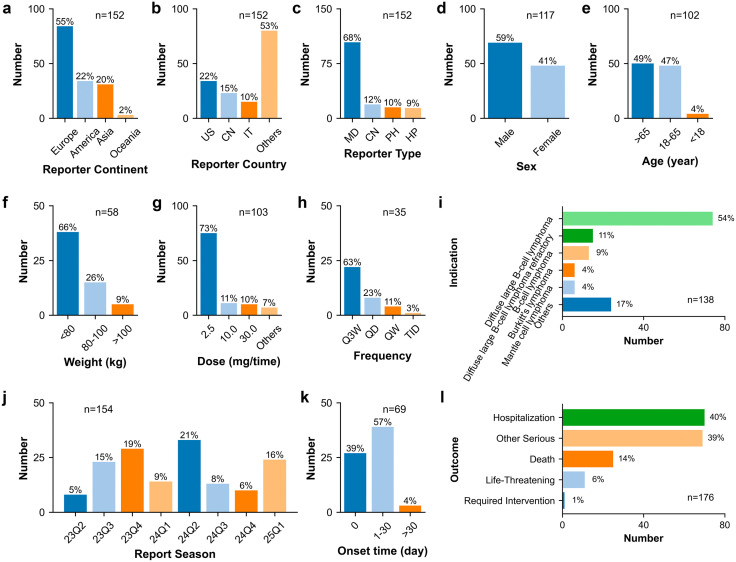
Clinical characteristics of glofitamab-associated cytokine release syndrome reports. **(a)** Reporter continent. **(b)** Reporter country. **(c)** Reporter type. **(d)** Sex. **(e)** Age. **(f)** Weight. **(g)** Dose. **(h)** Frequency. **(i)** Indication. **(j)** Report season. **(k)** Onset time. **(l)** Outcome. Abbreviations: US, United States; CN, China; IT: Italy; MD, Physician; CN, consumer; PH, pharmacist; HP, Health-professional; Q3W, once every 3 weeks; QD, once a day; QW, once a week; TID, three times a day.

**Fig 5:**
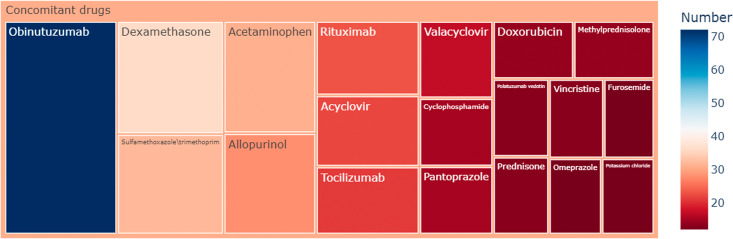
Rectangular tree plot of top 20 ranked concomitant drugs of glofitamab-associated cytokine release syndrome reports.

The clinical characteristics of 641 glofitamab-associated AE reports are shown in Figs 2 and 3. Regarding the continent of the reporters, this study included 638 valid reports, with Europe contributing the largest share at 46% (n = 293), followed by Asia at 35% (n = 223), America at 15% (n = 98), and Oceania at 4% (n = 24). The majority of reports originated from China at 30% (n = 189), trailed by the United States at 14% (n = 87), and France at 12% (n = 75). The clinical characteristics of 154 glofitamab-associated CRS reports are shown in Figs 4 and 5. While AE reports from Asia accounted for 35%, CRS reports from Asia accounted for only 20%. In Europe and the America, the proportion of CRS reports increased compared to all AE reports (Europe: 46% increased to 55%, America: 15% increased to 22%). Furthermore, Chinese AE reports topped the list among all countries at 30%, but CRS reports ranked second at 15%. These results suggest that there may be differences in the reporting of glofitamab-associated CRS across different continents. The findings suggest further research to support these results.

In terms of reporter type, out of the 638 entries, physicians constituted the largest group of reporters at 56% (n = 356), followed by consumers at 27% (n = 173), health-professionals at 9% (n = 57), and pharmacists at 8% (n = 52). The predominance of submissions from professionals (including physicians, health-professionals and pharmacists) suggests a certain level of data reliability. Consumer-reported AE cases made up 27%, whereas consumer-reported CRS cases accounted for only 12%. Due to the technical nature of CRS, it is often reported by professionals. These findings underscore the importance of enhancing education on CRS-related symptoms for non-professionals such as consumers.

### Clinical characteristics regarding patient

In terms of sex distribution, 540 valid AE cases were examined, with males representing 60% (n = 323) and females 40% (n = 217). Age data was accessible for 434 AE cases, showing an average age of 62.6 years. Most patients were over 65 years old (50%, n = 215) or within the 18–65 age range (49%, n = 213), while a small minority were below 18 years of age (1%, n = 6). The weight data displayed a broad range with only 166 valid reports, spanning from 19.9 to 181.3 kg, with 69% below 80 kg (n = 115), 23% falling within the 80–100 kg range (n = 39), and 7% exceeding 100 kg (n = 12).

Among the 641 reports, 334 AE reports involved glofitamab alone, while 307 reports indicated concomitant drug usage. Obinutuzumab, acetaminophen, and dexamethasone stood out as the primary concomitant drugs associated with glofitamab, accounting for 30% (n = 195), 12% (n = 80), and 12% (n = 76) respectively. Given the potential impact of concomitant drugs on AE signals, these findings suggest the necessity of additional sensitivity analyses to isolate the effects of concomitant drug usage.

### Clinical characteristics regarding product

Transitioning to dosage considerations, the primary dose was 2.5 mg, accounting for 48% (n = 195), followed by 30.0 mg at 39% (n = 158), 10.0 mg at 10% (n = 40), with other doses making up 4% (n = 15). The predominant frequency observed was Q3W, representing 71% (n = 72). Notably, CRS reports associated with a dose of 2.5 mg increased from 48% to 73%. These results suggest that CRS may be more inclined to occur at the 2.5 mg dose.

Among the 556 reported indications, the majority were linked to “Diffuse large B-cell lymphoma”, “Diffuse large B-cell lymphoma refractory”, “B-cell lymphoma”, “Mantle cell lymphoma”, and “Diffuse large B-cell lymphoma recurrent”. Given the potential impact of the original disease on AE signals, this study proceeded additional sensitivity analyses only including AE reports with indication of diffuse large B-cell lymphoma (including “Diffuse large B-cell lymphoma”, “Diffuse large B-cell lymphoma refractory”, “Diffuse large B-cell lymphoma recurrent”, “Diffuse large B-cell lymphoma stage IV”).

### Clinical characteristics regarding problem

The number of AE reports associated with glofitamab ranged from 32 to 153 occurrences per quarter. Regarding the onset time, 18% (n = 39) of cases exhibited symptoms on the day, followed by 44% (n = 93) within 1–30 days, and 38% (n = 81) after 30 days. CRS reports on the day of administration and within 1–30 days increased to 39% and 57%. These results suggest that CRS may be more inclined to occur within the first 30 days after dosing of glofitamab.

Out of the 641 reports, 509 instances resulted in 602 serious outcomes, categorized as “Death” at 35% (n = 208), “Other Serious” at 32% (n = 193), “Hospitalization” at 29% (n = 173), and “Life-Threatening” at 4% (n = 26), among others. The results indicate the necessity of in-depth research focusing on AEs associated with glofitamab to avoid serious consequences.

### Sensitivity analysis

The results of sensitivity analysis-restricted to reports without concomitant drug use and reports with diffuse large B-cell lymphoma as the indication-are presented in S6–S7 Tables. The results confirmed that the signal of CRS persisted independent of polypharmacy [n: 47, ROR (95% CI): 109.82 (81.49–148.00), PRR (χ2): 101.57 (4651.87), IC (IC025): 6.66 (4.60), EBGM (EBGM05): 100.88 (74.86)] and unrelated original diseases [n: 96, ROR (95% CI): 134.43 (108.83–166.05), PRR (χ2): 122.28 (11385.80), IC (IC025): 6.91 (5.45), EBGM (EBGM05): 120.49 (97.55)]. Given the higher reliability of reports from professionals, the stratified analysis based on only professionals, as shown in S8 Table, also revealed a significant signal of CRS [n: 133, ROR (95% CI): 99.37 (82.81–119.24), PRR (χ2): 88.11 (11224.42), IC (IC025): 6.43 (5.45), EBGM (EBGM05): 86.25 (71.87)]. The results of stratified analyses-by sex, age and continent-are presented in S9–S15 Tables. Significant signals for CRS were consistently identified across all stratifications, including males, females, patients aged 18–65 years, patients aged over 65 years, as well as patients from Europe, Asia, and the Americas. This consistency indicates a high level of reliability in the results.

Notably, the continent-stratified analysis revealed interesting regional variations. A particularly strong signal of CRS was observed in patients from America [n: 34, ROR (95% CI): 356.41 (247.34–513.58), PRR (χ2): 304.55 (10186.55), IC (IC025): 8.24 (4.45), EBGM (EBGM05): 301.45 (209.20)]. In contrast, a notably weaker signal was detected in patients from Asia [n: 28, ROR (95% CI): 22.76 (15.51–33.41), PRR (χ2): 21.66 (542.97), IC (IC025): 4.41 (3.09), EBGM (EBGM05): 21.28 (14.50)]. By conducting these stratified analyses, to some extent, potential reporting biases from different continents were partly mitigated. These results reveal that after using glofitamab, individuals from America exhibited a stronger signal for CRS compared to individuals from Asia, further suggest that differences may exist in the reporting of CRS across different continents.

### Signal differences detection

**Fig 6:**
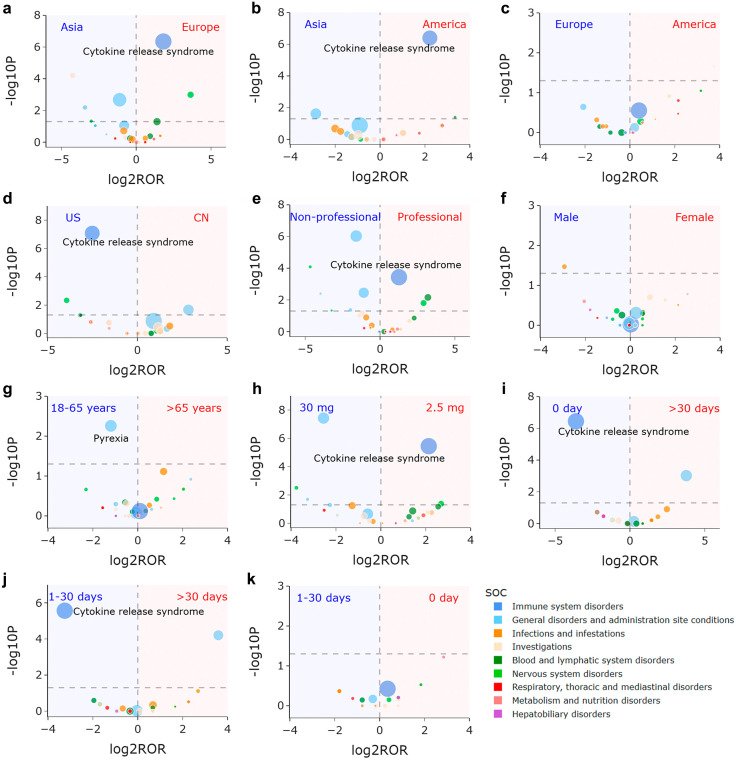
Volcano plots for difference detection of glofitamab signals across different subgroups. **(a)** Difference between Europe and Asia reporters. **(b)** Difference between America and Asia reporters. **(c)** Difference between America and Europe reporters. **(d)** Difference between China and United States reporters. **(e)** Difference between professional and non-professional reporters. **(f)** Difference between female and male patients. **(g)** Difference between patients with age > 65 years and 18-65 years. **(h)** Difference between patients taking signal dose 2.5 mg and 30 mg. **(i)** Difference between onset time >30 days and 0 day. **(j)** Difference between onset time >30 days and 1-30 days. **(k)** Difference between onset time 0 day and 1-30 days. The sizes of each point represent the number of reports of each PT induced by glofitamab. In these plots, 38 glofitamab-associated signals in Table 2 are shown. Abbreviations: US, United States; CN, China.

Volcano plots displaying glofitamab-related signal variances across different subgroups including reporter continent, reporter country, reporter type, sex, age, dose, and onset time are depicted in **Fig 6**. Since FAERS database was established by the United States FDA, all AEs were reported from the United States and mainly serious AEs were reported from other countries. To reduce reporting biases among different countries, in the subgroup analyses regarding the reporter continent and reporter country, only reports resulting in serious outcomes were included. Under this restriction, the statistical results still revealed significant regional disparities in CRS reporting. Compared to Asian reporters, both American and European reporters demonstrated significantly higher CRS reporting frequency [America vs. Asia: ROR (95% CI): 4.654 (2.452–8.836), P < 0.001; Europe vs. Asia: ROR (95% CI): 3.523 (2.104–5.901), P < 0.001]. Furthermore, when comparing China and United States (the two countries with the highest glofitamab-related AE reports), the United States showed significantly more frequent CRS reporting [China vs. United States: ROR (95% CI): 0.175 (0.087–0.353), P < 0.001]. While these differences might still reflect national reporting biases, when considered alongside findings from clinical characteristics and continent-stratified analysis, the findings suggest potential reporting differences in glofitamab-related AEs across different continents.

Professional reporters were significantly more frequently to report CRS than non-professional reporters [Professionals vs. non-professionals: ROR (95% CI): 2.401 (1.463–3.942), P < 0.001]. This finding aligns with the results in clinical characteristics, underscoring the urgent need to improve patient education programs, particularly regarding CRS recognition and associated psychological symptoms.

As for dosage, patients receiving 2.5 mg of glofitamab demonstrated significantly higher CRS reporting frequency compared to those on 30 mg [2.5 mg vs. 30 mg: ROR (95% CI): 4.388 (2.233–8.623), P < 0.001]. Onset time analysis revealed that CRS were reported more frequently on the day and within 1−30 days post-administration [>30 days vs. 0 day: ROR (95% CI): 0.082 (0.024–0.277), P < 0.001; > 30 days vs. 1−30 days: ROR (95% CI): 0.105 (0.032–0.345), P < 0.001]. These results, corroborating findings in clinical characteristics, emphasize the critical importance of intensive CRS monitoring for patients receiving the 2.5 mg dose and during the initial 30-day period following glofitamab administration.

## Discussion

Glofitamab is a T-cell-engaging bispecific monoclonal antibody being developed for the treatment of B-cell NHLs, including DLBCL. Nevertheless, the AEs reported in clinical trials might not completely reflect the long-term or rare AEs that could arise in clinical practice following the marketing of glofitamab. Furthermore, the differences in glofitamab-related AEs among subgroups such as reporter continent, reporter country, reporter type, sex, dose, and onset time are not yet fully elucidated. To address this limitation, the present study conducted a comprehensive analysis of AEs that were reported following the post-marketing period of glofitamab.

### Cytokine release syndrome

This study identified a total of 641 reported AEs, with 24 glofitamab-related SOCs. “Immune system disorders” was the most significant SOC that met four criteria. This emphasizes the importance of monitoring immune-related AEs, as they can have a significant impact on treatment outcomes and patient management strategies. This study identified CRS as a prominent AE. Dickinson et al. showed that CRS was observed in approximately 63% of patients who were treated with glofitamab [9]. Hutchings et al. suggested that CRS was observed in approximately 50.3% and 71.4% of patients from all glofitamab cohorts and RP2D glofitamab cohort, respectively [11]. The strong association between glofitamab and CRS is of particular concern, given that CRS can have severe clinical implications if not recognized and managed promptly. These findings highlight the need to improve the diagnosis and management of CRS when using glofitamab.

Based on the type of reporter, glofitamab-related AEs were predominantly reported by professionals, who demonstrate greater consistency and precision in categorization. And consumers only reported 27% of AEs and 12% of CRS. CRS can have serious clinical manifestations; therefore, educational programmes targeting consumers and healthcare providers alike are essential in facilitating the prompt identification of CRS symptoms, thereby improving patient safety [27].

This study also explored the differences in reporting AEs and CRS related to glofitamab across different continents. According to the reports, those from the Europe and Asia accounted for the vast majority, while those from America and Oceania accounted for a small proportion. This difference may due to the number of patients included in different continents. For example, in the STARGLO trial, Asia or Australia accounted for 59%, followed by Europe and North America accounted for 32% and 9%, respectively [28]. The reason fewer patients from America received glofitamab may be that highly effective follow-up therapies, such as chimeric antigen receptor T (CAR-T) cell therapy, are widely used for their relapsed/refractory DLBCL patients [29,30]. In terms of reporter country, China had the highest proportion of AEs reports among all nations, but CRS reports ranked second, after the United States. A retrospective study of 70 patients received glofitamab from 19 United States centers demonstrated any-grade CRS occurred in 28.6% of patients [31]. Another retrospective study of 69 patients with relapsed/refractory DLBCL received glofitamab monotherapy had CRS occurring in 46% (n = 31) [32]. Song et al. reported that all 30 relapsed/refractory DLBCL Chinese patients who received glofitamab monotherapy had AEs, including 63.3% cases of CRS with grade ≥3 occurred in 1 (3.3%) patient [12]. Cartron et al. suggested that 82.6% participants in France experienced at least one AE, and only 8.7% patients had CRS [30], which was much lower than previous studies [9,11]. This difference may due to French patients had experienced CAR-T cell therapy before glofitamab, with depleted peripheral lymphocyte [30]. Birtas Atesoglu et al. indicated that 12 (27.9%) cases developed CRS among 43 Turkish patients, grade ≥3 CRS was documented in 4 (9.3%) patients [33]. This study suggests that further research is needed to explore differences in glofitamab usage among different continents in a controlled setting.

According to the reports based on sex, the proportions of females and males were 40% and 60%, respectively. Zhou et al. [34] reported that males with DLBCL had worse outcomes with lower progression-free survival (PFS) than females, especially in the elderly age group (>60 years). Therefore, males may be overrepresented in relapsed/refractory DLBCL, which is consistent with previous studies [9,30].

In the present study, CRS was most frequently reported in patients who received a 2.5 mg dose of glofitamab. Glofitamab is administered using a step-up dosing regimen, and the 2.5 mg dose typically represents the first exposure, during which T cells are most likely to be activated, thereby triggering CRS. Donald et al. [35] reported that 56% of patients developed CRS after the first dose of glofitamab, compared to 35% and 29% after the second and third doses, respectively, with an incidence of 2.8% after subsequent doses. Furthermore, fatal CRS events, caused by an accumulation of the immune cells in local tissues and extensive production of cytokines, typically occurred after the initial glofitamab dose [9,33,36]. This phenomenon is more likely due to the dosing strategy and underlying immunological mechanisms. To reduce the risk of CRS, patients must strictly receive a single 1000-mg dose of obinutuzumab seven days before the first glofitamab dose for depletion of B cells in the peripheral blood and lymphoid tissue. In terms of the onset time, the highest proportion of CRS was reported within 1–30 days, followed by reports on the day of glofitamab administration, only 4% reported after 30 days. Studies by Dickinson et al. and Hutchings et al. [9,11] suggested that the median times to the earliest onset of CRS relative to the last prior glofitamab dose were 13.5 hours (range 6.0–52.0) and 10.8 hours (range 3.0–47.0) respectively, with median durations of 30.5 hours (range 0.5–317.0) and 2.2 days (range 0.0–31.0). Therefore, once glofitamab has been administered, healthcare professionals need to pay close attention to CRS, which may be serious or last for several days, especially with a dose of 2.5 mg.

### Hypogammaglobulinaemia and infections

This study identified 38 signals related to glofitamab, the majority of which were in line with the information provided on the drug label. A new significant signal was discovered, namely “hypogammaglobulinaemia”. The results of sensitivity analyses confirmed that the signal of hypogammaglobulinaemia persisted independent of polypharmacy and non-professional reports, indicates a high level of reliability in the results. However, when glofitamab-associated AE reports without diffuse large B-cell lymphoma as the treatment indication were excluded, the signal of hypogammaglobulinaemia became not-significant. The limited number of cases may account for its absence in previous trials, which may have led to the exclusion of these cases from the analysis. Currently, no studies have been found that link hypogammaglobulinaemia to glofitamab. Nevertheless, insights from studies on other bispecific antibodies may provide some context. Blinatumomab, a CD3/CD19-directed bispecific T-cell engager molecule with relapsed or refractory B-cell acute lymphoblastic leukemia (B-ALL), has been shown to result in B-cell aplasia and hence reduced immunoglobulin levels [37]. Mosunetuzumab, an anti-CD20/CD3 T-cell engaging bispecific antibody for the treatment of relapsed or refractory follicular lymphoma (FL), has been associated with a 40% incidence of hypogammaglobulinaemia [38]. The establishment of a definitive pharmacoepidemiological relationship between glofitamab and hypogammaglobulinaemia warrants further comprehensive pharmacovigilance monitoring.

In addition to CRS reactions, infection is another significant AE of glofitamab. Several studies revealed that infections occurred in 38–51.5% of cases, with the most frequently reported infection being SARS-CoV-2 (Covid-19), while the remaining infectious complications were not reported [9,11]. This study identified several PTs, including cytomegalovirus infection reactivation, pneumocystis jirovecii pneumonia, disseminated tuberculosis, as glofitamab-related signals. The role of the adaptive immune system in combatting infection through immunoglobulin function is well established. Hypogammaglobulinaemia related to an increased risk of infection and high severity of infections has been noted, especially when IgG levels remain depleted for more than 4 months [39]. While hypogammaglobulinaemia accompanied by lymphopenia, particularly CD4^+^ T-cell lymphopenia, opportunistic infection such as Mycobacterium tuberculosis, pneumocystis jirovecii pneumonia, cytomegalovirus, varicella-zoster virus (VZV) and BK virus can occur with an increased rate [40].

Healthcare professionals must pay close attention to the early signs of encapsulated bacterial organisms infections in patients with symptomatic hypogammaglobulinaemia, initiating prompt empiric antimicrobial therapy at the first sign of infection to minimize infectious morbidity and mortality [41]. And monthly replacement of intravenous immunoglobulins (IVIg) is indicated for IgG level <4 g/L or in the presence of recurrent severe bacterial infections [42]. These findings suggest the necessity to improve the diagnosis and management of hypogammaglobulinaemia and infections when using glofitamab.

### Strengths and limitations

This study has several strengths. Firstly, the FAERS database under consideration is the most exhaustive repository of post-marketing safety data for pharmaceuticals. Secondly, this study explored the differences in AE signals concerning continent, country, reporter type, sex, dose and onset time. Thirdly, in addition to the AEs listed in the drug insert and clinical trials, this study identified a new significant AE, namely “hypogammaglobulinemia”. It is hoped that this will serve as a valuable and comprehensive reference for the safety study of glofitamab.

It is imperative to acknowledge the several limitations of this study. Firstly, FAERS is a spontaneous reporting system and introduces variability in data quality owing to the heterogeneity of its sources. Secondly, FAERS lacks data on the total number of patients treated with glofitamab, which makes it impossible to quantify the incidence of each AE. Thirdly, the voluntary nature of the reporting system may result in under-reporting, bias and a lack of control group. Fourthly, apart from factors like dose and onset time, there are still various influencing factors for glofitamab-related AEs, such as indication, and concomitant medication. Although concomitant medication like traditional Chinese medicines have demonstrated anti-tumor, anti-infective and immunomodulation properties [43–47], this study did not account for these potential confounding factors. A phase I/II study of glofitamab in relapsed/refractory mantle cell lymphoma (MCL) suggested that CRS occurred in 70% of patients, and with a lower incidence in the 2,000 mg obinutuzumab, compared to 1,000 mg [48]. Despite these limitations, this study provides healthcare professionals with valuable insights into monitoring patients and AEs associated with glofitamab.

## Conclusion

This study conducted a comprehensive analysis of glofitamab using the FAERS database. The findings suggest potential reporting differences in glofitamab-related AEs across different continents. Educating consumers on how to recognize the early symptoms of CRS is essential to improve safety. Close monitoring of CRS is recommended within 30 days of administration of glofitamab, especially at a dose of 2.5 mg. Furthermore, it is essential to stay vigilant about the emergence of the newly identified AE. These findings contribute to a broader understanding of the AE profiles of glofitamab.

## Supporting information

S1 Table Fourfold table of disproportionality analysis for glofitamab signal detection. (DOCX)

S2 Table Equations and criteria of four algorithms for glofitamab signal detection. (DOCX)

S3 Table Fourfold table of disproportionality analysis for difference detection of glofitamab signals. (DOCX)

S4 Table Criteria of ROR and Chi-Square Test/Fisher’s exact Test for difference detection of glofitamab signals. (DOCX)

S5 Table Number and signal strength of 10 glofitamab-unrelated signals at the PT level. (DOCX)

S6 Table Number and signal strength of glofitamab-related signals at the PT level based on reports without concomitant drugs use. (DOCX)

S7 Table Number and signal strength of glofitamab-related signals at the PT level based on reports with diffuse large B-cell lymphoma indication. (DOCX)

S8 Table Number and signal strength of glofitamab-related signals at the PT level stratified by professionals. (DOCX)

S9 Table Number and signal strength of glofitamab-related signals at the PT level stratified by males. (DOCX)

S10 Table Number and signal strength of glofitamab-related signals at the PT level stratified by females. (DOCX)

S11 Table Number and signal strength of glofitamab-related signals at the PT level stratified by patients aged 18–65 years. (DOCX)

S12 Table Number and signal strength of glofitamab-related signals at the PT level stratified by patients aged >65 years. (DOCX)

S13 Table Number and signal strength of glofitamab-related signals at the PT level stratified by Europe patients. (DOCX)

S14 Table Number and signal strength of glofitamab-related signals at the PT level stratified by Asia patients. (DOCX)

S15 Table Number and signal strength of glofitamab-related signals at the PT level stratified by America patients. (DOCX)

## Abbreviations

AE: adverse event
B-ALL: B-cell acute lymphoblastic leukemia
BCPNN: Bayesian confidence propagation neural network
CAR-T: chimeric antigen receptor T
CRS: cytokine release syndrome
DLBCL: diffuse large B-cell lymphoma
FAERS: the FDA Adverse Event Reporting System
FL: follicular lymphoma
IVIg: intravenous immunoglobulins
MCL: mantle cell lymphoma
MGPS: multi-item gamma Poisson shrinker
NHL: non-Hodgkin lymphoma
PFS: progression-free survival
PRR: proportional reporting ratio
PS: primary suspect
PT: preferred term
ROR: reporting odds ratio
SOC: system organ class
VZV: varicella-zoster virus
