# Supplementary material for: Exploration of adverse event profiles for glofitamab: A disproportionality analysis using the FDA adverse event reporting system
Source: PLoS One. 2025 Nov 4;20(11):e0336151. doi: 10.1371/journal.pone.0336151 (PMC12585042; doi:10.1371/journal.pone.0336151)
Supplement: S1 Table — (DOCX) [file pone.0336151.s001.docx]

**S1 Table. Fourfold table of disproportionality analysis for glofitamab signal detection.**

|  | Target adverse event | Other adverse events | Total |
| --- | --- | --- | --- |
| Glofitamab | a | b | a+b |
| Other drugs | c | d | c+d |
| Total | a+c | b+d | N=a+b+c+d |

For specific details, please refer to the references cited in the Method section of the manuscript.
