# Supplementary material for: Exploration of adverse event profiles for glofitamab: A disproportionality analysis using the FDA adverse event reporting system
Source: PLoS One. 2025 Nov 4;20(11):e0336151. doi: 10.1371/journal.pone.0336151 (PMC12585042; doi:10.1371/journal.pone.0336151)
Supplement: S2 Table — (DOCX) [file pone.0336151.s002.docx]

**S2 Table. Equations and criteria of four algorithms for glofitamab signal detection.**

| Algorithms | Equation | Criteria |
| --- | --- | --- |
| ROR | ROR=(ad)/(bc) | Lower limit of 95% CI>1, N≥2 |
|  | Lower limit of 95% CI=e^ln(ROR)-1.96(1/a+1/b+1/c+1/d)^0.5^  Upper limit of 95% CI=e^ln(ROR)+1.96(1/a+1/b+1/c+1/d)^0.5^ |  |
| PRR | PRR=a(c+d)/c/(a+b) | PRR≥2, χ2≥4, N≥3 |
|  | χ2=[(ad-bc)^2](a+b+c+d)/[(a+b)(c+d)(a+c)(b+d)] |  |
| BCPNN | IC=log_2_a(a+b+c+d)/(a+c)/(a+b) | IC025>0 |
|  | IC025=E(IC)-2V(IC)^0.5 |  |
| MGPS | EBGM=a(a+b+c+d)/(a+c)/(a+b) | EBGM05>2 |
|  | EBGM05=e^ln(EBGM)-1.96(1/a+1/b+1/c+1/d)^0.5^ |  |

**Abbreviations:** ROR, reporting odds ratio; CI, confidence interval; PRR, proportional reporting ratio; χ2, chi-squared; BCPNN, Bayesian confidence propagation neural network; IC, information component; IC025, lower limit of 95% confidence interval of IC; E(IC), IC expectation; V(IC), variance of IC; MGPS, multi-item gamma Poisson shrinker; EBGM, empirical Bayesian geometric mean; EBGM05, lower limit of 95% confidence interval of EBGM. For specific details, please refer to the references cited in the Method section of the manuscript.
