# Supplementary material for: Exploration of adverse event profiles for glofitamab: A disproportionality analysis using the FDA adverse event reporting system
Source: PLoS One. 2025 Nov 4;20(11):e0336151. doi: 10.1371/journal.pone.0336151 (PMC12585042; doi:10.1371/journal.pone.0336151)
Supplement: S4 Table — (DOCX) [file pone.0336151.s004.docx]

**S4 Table**. **Criteria of ROR and Chi-Square Test/Fisher’s exact Test for difference detection of glofitamab signals.**

|  | ROR | Chi-Square Test/Fisher’s exact Test |
| --- | --- | --- |
| Signals that subgroup 1 more frequently to report | ROR>1, Lower limit of 95% CI>1,  N≥2 | P<0.05 |
| Signals that subgroup 2 more frequently to report | ROR<1, Upper limit of 95% CI<1,  N≥2 | P<0.05 |

**Abbreviations:** ROR, reporting odds ratio; 95% CI, 95% confidence interval. When N is greater than or equal to 40 and a, b, c, and d are all greater than or equal to 5, Chi-Square Test is used. When N is less than or equal to 40 or one of a, b, c, d is less than 5, the Fisher’s exact Test is applied. For specific details, please refer to the references cited in the Method section of the manuscript.
