# Supplementary material for: Exploration of adverse event profiles for glofitamab: A disproportionality analysis using the FDA adverse event reporting system
Source: PLoS One. 2025 Nov 4;20(11):e0336151. doi: 10.1371/journal.pone.0336151 (PMC12585042; doi:10.1371/journal.pone.0336151)
Supplement: S5 Table — (DOCX) [file pone.0336151.s005.docx]

**S5 Table .** **Number and signal strength of** **10 glofitamab-unrelated signals at the PT level.**

| **PT** | **Number** | **ROR (95% CI)** | **PRR (χ2)** | **IC (IC025)** | **EBGM (EBGM05)** |
| --- | --- | --- | --- | --- | --- |
| **Neoplasms benign, malignant and unspecified (incl cysts and polyps) (SOC: 10029104)** | | | | | |
| Tumour flare (PT: 10045169) | 15 | 918.41 (532.14-1585.05) | 909.48 (11805.47) | 9.62 (3.20) | 788.89 (457.10) |
| Diffuse large B-cell lymphoma recurrent (PT: 10012821) | 9 | 98.88 (51.07-191.42) | 98.30 (852.78) | 6.60 (2.27) | 96.72 (49.96) |
| Neoplasm progression (PT: 10061309) | 6 | 5.05 (2.26-11.26) | 5.03 (19.40) | 2.33 (0.58) | 5.03 (2.26) |
| Lymphoma (PT: 10025310) | 5 | 16.13 (6.70-38.87) | 16.09 (70.56) | 4.00 (1.01) | 16.04 (6.66) |
| Acute myeloid leukaemia (PT: 10000880) | 4 | 10.92 (4.09-29.17) | 10.90 (35.90) | 3.44 (0.58) | 10.88 (4.07) |
| Myelodysplastic syndrome (PT: 10028533) | 4 | 13.45 (5.04-35.93) | 13.42 (45.89) | 3.74 (0.65) | 13.39 (5.01) |
| Neoplasm (PT: 10028980) | 3 | 9.65 (3.11-29.97) | 9.63 (23.17) | 3.27 (0.16) | 9.62 (3.10) |
| Diffuse large B-cell lymphoma (PT: 10012818) | 3 | 10.42 (3.36-32.39) | 10.41 (25.47) | 3.38 (0.19) | 10.39 (3.34) |
| **Others** | | | | | |
| Disease progression (PT: 10061818) | 83 | 25.15 (20.15-31.39) | 23.85 (1814.21) | 4.57 (3.90) | 23.76 (19.04) |
| No adverse event (PT: 10067482) | 30 | 4.06 (2.83-5.83) | 4.00 (67.92) | 2.00 (1.34) | 4.00 (2.79) |

**Abbreviations:** PT, preferred term; ROR, reporting odds ratio; CI, confidence interval; PRR, proportional reporting ratio; χ2, chi-squared; IC, information component; IC025, lower limit of 95% confidence interval of IC; EBGM, empirical Bayesian geometric mean; EBGM05, lower limit of 95% confidence interval of EBGM.
