# Supplementary material for: Exploration of adverse event profiles for glofitamab: A disproportionality analysis using the FDA adverse event reporting system
Source: PLoS One. 2025 Nov 4;20(11):e0336151. doi: 10.1371/journal.pone.0336151 (PMC12585042; doi:10.1371/journal.pone.0336151)
Supplement: S6 Table — (DOCX) [file pone.0336151.s006.docx]

**S6 Table.** **Number and signal strength of glofitamab-related signals at the PT level based on** **reports without concomitant drugs use.**

| **PT** | **Number** | **ROR (95% CI)** | **PRR (χ2)** | **IC (IC025)** | **EBGM (EBGM05)** |
| --- | --- | --- | --- | --- | --- |
| **General disorders and administration site conditions (SOC: 10018065)** | | | | | |
| Death (PT: 10011906) | 57 | 7.72 (5.88-10.14) | 7.11 (302.86) | 2.83 (2.29) | 7.10 (5.41) |
| Pyrexia (PT: 10037660) | 37 | 11.88 (8.52-16.56) | 11.23 (346.37) | 3.49 (2.66) | 11.22 (8.05) |
| Hyperpyrexia (PT: 10020741) | 7 | 143.33 (67.80-302.99) | 141.72 (968.90) | 7.13 (1.90) | 140.39 (66.41) |
| Organ failure (PT: 10053159) | 4 | 154.55 (57.53-415.19) | 153.56 (600.04) | 7.25 (0.98) | 151.99 (56.57) |
| **Immune system disorders (SOC: 10021428)** | | | | | |
| Cytokine release syndrome (PT: 10052015) | 47 | 109.82 (81.49-148.00) | 101.57 (4651.87) | 6.66 (4.60) | 100.88 (74.86) |
| Hypogammaglobulinaemia (PT: 10020983) | 3 | 29.10 (9.35-90.57) | 28.96 (80.84) | 4.85 (0.41) | 28.91 (9.29) |
| **Infections and infestations (SOC: 10021881)** | | | | | |
| Pneumonia (PT: 10035664) | 12 | 3.63 (2.05-6.43) | 3.58 (22.43) | 1.84 (0.77) | 3.58 (2.02) |
| Infection (PT: 10021789) | 8 | 4.86 (2.42-9.77) | 4.81 (24.23) | 2.27 (0.79) | 4.81 (2.40) |
| Septic shock (PT: 10040070) | 3 | 6.77 (2.18-21.06) | 6.74 (14.68) | 2.75 (0.02) | 6.74 (2.17) |
| Disseminated tuberculosis (PT: 10013453) | 3 | 132.60 (42.43-414.35) | 131.96 (386.47) | 7.03 (0.51) | 130.80 (41.86) |
| **Nervous system disorders (SOC: 10029205)** | | | | | |
| Immune effector cell-associated neurotoxicity syndrome (PT: 10083347) | 9 | 54.74 (28.31-105.84) | 53.96 (466.25) | 5.75 (2.18) | 53.77 (27.81) |
| Movement disorder (PT: 10028035) | 7 | 24.84 (11.79-52.36) | 24.57 (158.12) | 4.62 (1.61) | 24.54 (11.64) |
| Neurotoxicity (PT: 10029350) | 4 | 21.17 (7.91-56.62) | 21.04 (76.26) | 4.39 (0.77) | 21.01 (7.85) |
| Cerebral haemorrhage (PT: 10008111) | 3 | 11.76 (3.78-36.59) | 11.71 (29.38) | 3.55 (0.22) | 11.70 (3.76) |
| **Investigations (SOC: 10022891)** | | | | | |
| Platelet count decreased (PT: 10035528) | 9 | 8.28 (4.29-15.99) | 8.17 (56.72) | 3.03 (1.33) | 8.17 (4.23) |
| **Metabolism and nutrition disorders (SOC: 10027433)** | | | | | |
| Feeding disorder (PT: 10061148) | 3 | 11.11 (3.57-34.55) | 11.06 (27.43) | 3.47 (0.20) | 11.05 (3.55) |

In this sensitivity analysis, the AE reports of glofitamab without concomitant drugs use were included. **Abbreviations:** PT, preferred term; ROR, reporting odds ratio; CI, confidence interval; PRR, proportional reporting ratio; χ2, chi-squared; IC, information component; IC025, lower limit of 95% confidence interval of IC; EBGM, empirical Bayesian geometric mean; EBGM05, lower limit of 95% confidence interval of EBGM.
