# Supplementary material for: Exploration of adverse event profiles for glofitamab: A disproportionality analysis using the FDA adverse event reporting system
Source: PLoS One. 2025 Nov 4;20(11):e0336151. doi: 10.1371/journal.pone.0336151 (PMC12585042; doi:10.1371/journal.pone.0336151)
Supplement: S7 Table — (DOCX) [file pone.0336151.s007.docx]

**S7 Table.** **Number and signal strength of glofitamab-related signals at the PT level based on reports** **with** **diffuse large B-cell lymphoma indication.**

| **PT** | **Number** | **ROR (95% CI)** | **PRR (χ2)** | **IC (IC025)** | **EBGM (EBGM05)** |
| --- | --- | --- | --- | --- | --- |
| **General disorders and administration site conditions (SOC: 10018065)** | | | | | |
| Death (PT: 10011906) | 61 | 4.69 (3.62-6.07) | 4.47 (166.59) | 2.16 (1.70) | 4.47 (3.45) |
| Pyrexia (PT: 10037660) | 48 | 8.93 (6.69-11.93) | 8.57 (322.41) | 3.10 (2.47) | 8.56 (6.41) |
| Hyperpyrexia (PT: 10020741) | 7 | 83.91 (39.76-177.08) | 83.36 (564.26) | 6.37 (1.85) | 82.58 (39.13) |
| Organ failure (PT: 10053159) | 4 | 90.66 (33.79-243.25) | 90.32 (349.72) | 6.48 (0.96) | 89.40 (33.32) |
| Multiple organ dysfunction syndrome (PT: 10077361) | 4 | 6.33 (2.37-16.90) | 6.31 (17.87) | 2.66 (0.32) | 6.30 (2.36) |
| **Immune system disorders (SOC: 10021428)** | | | | | |
| Cytokine release syndrome (PT: 10052015) | 96 | 135.13 (109.41-166.90) | 122.91 (11455.20) | 6.92 (5.45) | 121.21 (98.14) |
| **Investigations (SOC: 10022891)** | | | | | |
| Platelet count decreased (PT: 10035528) | 15 | 8.12 (4.87-13.51) | 8.01 (92.16) | 3.00 (1.75) | 8.01 (4.81) |
| Alanine aminotransferase increased (PT: 10001551) | 11 | 14.07 (7.76-25.50) | 13.93 (131.95) | 3.80 (1.91) | 13.91 (7.68) |
| White blood cell count decreased (PT: 10047942) | 9 | 4.71 (2.44-9.08) | 4.68 (26.07) | 2.23 (0.86) | 4.68 (2.43) |
| Blood lactate dehydrogenase increased (PT: 10005630) | 7 | 37.83 (17.96-79.68) | 37.59 (248.27) | 5.23 (1.73) | 37.43 (17.77) |
| Aspartate aminotransferase increased (PT: 10003481) | 7 | 10.45 (4.97-21.98) | 10.39 (59.35) | 3.38 (1.23) | 10.38 (4.93) |
| Blood bilirubin increased (PT: 10005364) | 6 | 18.36 (8.22-41.00) | 18.26 (97.74) | 4.19 (1.30) | 18.23 (8.16) |
| Liver function test increased (PT: 10077692) | 4 | 7.61 (2.85-20.33) | 7.59 (22.88) | 2.92 (0.42) | 7.58 (2.84) |
| **Blood and lymphatic system disorders (SOC: 10005329)** | | | | | |
| Neutropenia (PT: 10029354) | 21 | 7.57 (4.91-11.66) | 7.44 (117.21) | 2.89 (1.90) | 7.43 (4.82) |
| Anaemia (PT: 10002034) | 16 | 5.89 (3.59-9.65) | 5.81 (63.87) | 2.54 (1.47) | 5.81 (3.54) |
| Thrombocytopenia (PT: 10043554) | 13 | 7.14 (4.13-12.34) | 7.06 (67.72) | 2.82 (1.52) | 7.06 (4.08) |
| Leukocytosis (PT: 10024378) | 3 | 11.70 (3.77-36.38) | 11.67 (29.25) | 3.54 (0.22) | 11.66 (3.75) |
| **Infections and infestations (SOC: 10021881)** | | | | | |
| Infection (PT: 10021789) | 15 | 5.37 (3.23-8.95) | 5.31 (52.58) | 2.41 (1.34) | 5.31 (3.19) |
| Septic shock (PT: 10040070) | 11 | 14.71 (8.12-26.65) | 14.56 (138.83) | 3.86 (1.93) | 14.54 (8.02) |
| Herpes zoster (PT: 10019974) | 5 | 5.20 (2.16-12.53) | 5.18 (16.89) | 2.37 (0.43) | 5.18 (2.15) |
| Urosepsis (PT: 10048709) | 3 | 20.87 (6.71-64.91) | 20.81 (56.46) | 4.38 (0.36) | 20.77 (6.68) |
| **Nervous system disorders (SOC: 10029205)** | | | | | |
| Immune effector cell-associated neurotoxicity syndrome (PT: 10083347) | 12 | 42.85 (24.22-75.80) | 42.37 (482.54) | 5.40 (2.53) | 42.17 (23.84) |
| Movement disorder (PT: 10028035) | 7 | 14.54 (6.91-30.60) | 14.45 (87.56) | 3.85 (1.40) | 14.43 (6.86) |
| Neurotoxicity (PT: 10029350) | 4 | 12.42 (4.65-33.17) | 12.38 (41.78) | 3.63 (0.62) | 12.36 (4.63) |
| Cerebral haemorrhage (PT: 10008111) | 4 | 9.22 (3.45-24.62) | 9.19 (29.17) | 3.20 (0.51) | 9.18 (3.44) |
| **Metabolism and nutrition disorders (SOC: 10027433)** | | | | | |
| Hypophosphataemia (PT: 10021058) | 5 | 36.24 (15.03-87.41) | 36.07 (169.82) | 5.17 (1.21) | 35.93 (14.90) |
| Feeding disorder (PT: 10061148) | 3 | 6.52 (2.10-20.26) | 6.50 (13.97) | 2.70 (0.01) | 6.50 (2.09) |
| Tumour lysis syndrome (PT: 10045170) | 3 | 17.41 (5.60-54.13) | 17.36 (46.18) | 4.12 (0.32) | 17.33 (5.57) |
| **Respiratory, thoracic and mediastinal disorders (SOC: 10038738)** | | | | | |
| Respiratory failure (PT: 10038695) | 6 | 6.48 (2.90-14.45) | 6.45 (27.61) | 2.69 (0.76) | 6.44 (2.89) |
| Pneumonitis (PT: 10035742) | 4 | 7.31 (2.74-19.52) | 7.29 (21.69) | 2.86 (0.40) | 7.28 (2.73) |
| **Renal and urinary disorders (SOC: 10038359)** | | | | | |
| Haematuria (PT: 10018867) | 3 | 7.96 (2.56-24.75) | 7.94 (18.20) | 2.99 (0.09) | 7.94 (2.55) |

In this sensitivity analysis, the AE reports of glofitamab with indication of diffuse large B-cell lymphoma (including “Diffuse large B-cell lymphoma”, “Diffuse large B-cell lymphoma refractory”, “Diffuse large B-cell lymphoma recurrent”, “Diffuse large B-cell lymphoma stage IV”) were included. **Abbreviations:** PT, preferred term; ROR, reporting odds ratio; CI, confidence interval; PRR, proportional reporting ratio; χ2, chi-squared; IC, information component; IC025, lower limit of 95% confidence interval of IC; EBGM, empirical Bayesian geometric mean; EBGM05, lower limit of 95% confidence interval of EBGM.
