# Supplementary material for: Exploration of adverse event profiles for glofitamab: A disproportionality analysis using the FDA adverse event reporting system
Source: PLoS One. 2025 Nov 4;20(11):e0336151. doi: 10.1371/journal.pone.0336151 (PMC12585042; doi:10.1371/journal.pone.0336151)
Supplement: S8 Table — (DOCX) [file pone.0336151.s008.docx]

**S8 Table.** **Number and signal strength of glofitamab-related signals at the PT level** **stratified by professionals.**

| **PT** | **Number** | **ROR (95% CI)** | **PRR (χ2)** | **IC (IC025)** | **EBGM (EBGM05)** |
| --- | --- | --- | --- | --- | --- |
| **Immune system disorders (SOC: 10021428)** | | | | | |
| Cytokine release syndrome (PT: 10052015) | 133 | 99.37 (82.81-119.24) | 88.11 (11224.42) | 6.43 (5.45) | 86.25 (71.87) |
| Hypogammaglobulinaemia (PT: 10020983) | 5 | 14.14 (5.87-34.09) | 14.09 (60.59) | 3.81 (0.96) | 14.04 (5.82) |
| **General disorders and administration site conditions (SOC: 10018065)** | | | | | |
| Death (PT: 10011906) | 41 | 3.16 (2.32-4.32) | 3.09 (58.47) | 1.63 (1.10) | 3.09 (2.26) |
| Pyrexia (PT: 10037660) | 38 | 5.26 (3.81-7.27) | 5.12 (126.67) | 2.35 (1.74) | 5.12 (3.70) |
| Multiple organ dysfunction syndrome (PT: 10077361) | 6 | 4.92 (2.20-10.98) | 4.90 (18.61) | 2.29 (0.56) | 4.89 (2.19) |
| Hyperpyrexia (PT: 10020741) | 4 | 27.71 (10.35-74.22) | 27.62 (101.95) | 4.78 (0.83) | 27.44 (10.25) |
| Temperature intolerance (PT: 10057040) | 3 | 15.64 (5.02-48.66) | 15.60 (40.83) | 3.96 (0.30) | 15.54 (4.99) |
| **Infections and infestations (SOC: 10021881)** | | | | | |
| COVID-19 (PT: 10084268) | 25 | 3.92 (2.64-5.82) | 3.86 (53.12) | 1.95 (1.22) | 3.85 (2.59) |
| Infection (PT: 10021789) | 15 | 4.29 (2.58-7.15) | 4.25 (37.35) | 2.09 (1.09) | 4.25 (2.55) |
| Septic shock (PT: 10040070) | 11 | 7.87 (4.34-14.26) | 7.80 (65.19) | 2.96 (1.48) | 7.79 (4.30) |
| Cytomegalovirus infection reactivation (PT: 10058666) | 4 | 10.91 (4.08-29.15) | 10.87 (35.78) | 3.44 (0.57) | 10.85 (4.06) |
| COVID-19 pneumonia (PT: 10084380) | 4 | 7.28 (2.73-19.46) | 7.26 (21.57) | 2.86 (0.39) | 7.25 (2.71) |
| Disseminated tuberculosis (PT: 10013453) | 3 | 38.57 (12.35-120.41) | 38.47 (108.47) | 5.25 (0.44) | 38.12 (12.21) |
| **Blood and lymphatic system disorders (SOC: 10005329)** | | | | | |
| Neutropenia (PT: 10029354) | 28 | 5.29 (3.63-7.69) | 5.18 (94.85) | 2.37 (1.63) | 5.18 (3.56) |
| Thrombocytopenia (PT: 10043554) | 15 | 4.23 (2.54-7.04) | 4.19 (36.50) | 2.07 (1.08) | 4.19 (2.51) |
| Anaemia (PT: 10002034) | 15 | 3.56 (2.14-5.92) | 3.52 (27.20) | 1.82 (0.88) | 3.52 (2.12) |
| Leukocytosis (PT: 10024378) | 3 | 6.62 (2.13-20.58) | 6.61 (14.26) | 2.72 (0.01) | 6.60 (2.12) |
| **Investigations (SOC: 10022891)** | | | | | |
| Alanine aminotransferase increased (PT: 10001551) | 12 | 8.92 (5.05-15.77) | 8.84 (83.37) | 3.14 (1.66) | 8.82 (4.99) |
| Platelet count decreased (PT: 10035528) | 11 | 5.11 (2.82-9.25) | 5.07 (35.94) | 2.34 (1.08) | 5.06 (2.79) |
| Aspartate aminotransferase increased (PT: 10003481) | 8 | 6.91 (3.45-13.87) | 6.87 (40.12) | 2.78 (1.09) | 6.86 (3.42) |
| Blood lactate dehydrogenase increased (PT: 10005630) | 7 | 20.23 (9.60-42.61) | 20.11 (126.54) | 4.32 (1.54) | 20.02 (9.50) |
| Blood bilirubin increased (PT: 10005364) | 6 | 11.08 (4.96-24.75) | 11.03 (54.62) | 3.46 (1.08) | 11.01 (4.93) |
| SARS-CoV-2 test positive (PT: 10084271) | 4 | 11.34 (4.24-30.32) | 11.31 (37.49) | 3.50 (0.59) | 11.28 (4.22) |
| **Nervous system disorders (SOC: 10029205)** | | | | | |
| Immune effector cell-associated neurotoxicity syndrome (PT: 10083347) | 23 | 42.77 (28.25-64.77) | 41.95 (910.34) | 5.38 (3.35) | 41.53 (27.43) |
| Neurotoxicity (PT: 10029350) | 7 | 11.23 (5.34-23.63) | 11.17 (64.66) | 3.48 (1.27) | 11.14 (5.29) |
| **Metabolism and nutrition disorders (SOC: 10027433)** | | | | | |
| Tumour lysis syndrome (PT: 10045170) | 7 | 20.37 (9.67-42.91) | 20.26 (127.54) | 4.33 (1.54) | 20.16 (9.57) |
| Hypophosphataemia (PT: 10021058) | 5 | 19.45 (8.06-46.91) | 19.37 (86.69) | 4.27 (1.07) | 19.28 (7.99) |
| **Respiratory, thoracic and mediastinal disorders (SOC: 10038738)** | | | | | |
| Hypoxia (PT: 10021143) | 5 | 5.19 (2.16-12.51) | 5.18 (16.84) | 2.37 (0.43) | 5.17 (2.15) |
| Tachypnoea (PT: 10043089) | 3 | 7.17 (2.31-22.29) | 7.15 (15.86) | 2.84 (0.05) | 7.14 (2.30) |
| **Hepatobiliary disorders (SOC: 10019805)** | | | | | |
| Hypertransaminasaemia (PT: 10068237) | 4 | 8.51 (3.19-22.74) | 8.49 (26.37) | 3.08 (0.47) | 8.47 (3.17) |

In this stratified analysis, for both glofitamab and all other drugs, only reports submitted by professionals were included. **Abbreviations:** PT, preferred term; ROR, reporting odds ratio; CI, confidence interval; PRR, proportional reporting ratio; χ2, chi-squared; IC, information component; IC025, lower limit of 95% confidence interval of IC; EBGM, empirical Bayesian geometric mean; EBGM05, lower limit of 95% confidence interval of EBGM.
