# Supplementary material for: Exploration of adverse event profiles for glofitamab: A disproportionality analysis using the FDA adverse event reporting system
Source: PLoS One. 2025 Nov 4;20(11):e0336151. doi: 10.1371/journal.pone.0336151 (PMC12585042; doi:10.1371/journal.pone.0336151)
Supplement: S9 Table — (DOCX) [file pone.0336151.s009.docx]

**S9 Table.** **Number and signal strength of glofitamab-related signals at the PT level** **stratified by males.**

| **PT** | **Number** | **ROR (95% CI)** | **PRR (χ2)** | **IC (IC025)** | **EBGM (EBGM05)** |
| --- | --- | --- | --- | --- | --- |
| **Immune system disorders (SOC: 10021428)** | | | | | |
| Cytokine release syndrome (PT: 10052015) | 69 | 88.13 (68.65-113.14) | 80.27 (5293.76) | 6.30 (4.86) | 78.60 (61.23) |
| **General disorders and administration site conditions (SOC: 10018065)** | | | | | |
| Pyrexia (PT: 10037660) | 36 | 8.52 (6.09-11.91) | 8.16 (227.10) | 3.03 (2.28) | 8.15 (5.83) |
| Hyperpyrexia (PT: 10020741) | 5 | 60.72 (25.02-147.34) | 60.33 (287.13) | 5.89 (1.27) | 59.39 (24.47) |
| Multiple organ dysfunction syndrome (PT: 10077361) | 4 | 5.75 (2.15-15.38) | 5.73 (15.61) | 2.52 (0.26) | 5.72 (2.14) |
| Organ failure (PT: 10053159) | 3 | 71.98 (22.91-226.11) | 71.70 (205.21) | 6.14 (0.47) | 70.37 (22.40) |
| **Investigations (SOC: 10022891)** | | | | | |
| Platelet count decreased (PT: 10035528) | 8 | 5.21 (2.59-10.46) | 5.16 (26.87) | 2.37 (0.85) | 5.16 (2.57) |
| Aspartate aminotransferase increased (PT: 10003481) | 5 | 9.29 (3.85-22.41) | 9.24 (36.67) | 3.20 (0.77) | 9.22 (3.82) |
| Alanine aminotransferase increased (PT: 10001551) | 5 | 8.26 (3.42-19.91) | 8.21 (31.61) | 3.03 (0.71) | 8.19 (3.40) |
| Blood bilirubin increased (PT: 10005364) | 3 | 9.89 (3.18-30.78) | 9.86 (23.82) | 3.30 (0.17) | 9.83 (3.16) |
| **Nervous system disorders (SOC: 10029205)** | | | | | |
| Immune effector cell-associated neurotoxicity syndrome (PT: 10083347) | 11 | 37.21 (20.46-67.67) | 36.68 (378.26) | 5.18 (2.36) | 36.34 (19.98) |
| Movement disorder (PT: 10028035) | 5 | 14.29 (5.92-34.48) | 14.20 (61.14) | 3.82 (0.96) | 14.15 (5.86) |
| Neurotoxicity (PT: 10029350) | 3 | 9.35 (3.00-29.08) | 9.31 (22.22) | 3.22 (0.15) | 9.29 (2.99) |
| **Blood and lymphatic system disorders (SOC: 10005329)** | | | | | |
| Neutropenia (PT: 10029354) | 15 | 6.12 (3.67-10.20) | 6.02 (62.83) | 2.59 (1.46) | 6.01 (3.60) |
| **Infections and infestations (SOC: 10021881)** | | | | | |
| Septic shock (PT: 10040070) | 11 | 13.78 (7.59-25.01) | 13.59 (128.01) | 3.76 (1.89) | 13.55 (7.46) |
| Herpes zoster (PT: 10019974) | 4 | 7.56 (2.83-20.20) | 7.52 (22.59) | 2.91 (0.41) | 7.51 (2.81) |
| **Metabolism and nutrition disorders (SOC: 10027433)** | | | | | |
| Tumour lysis syndrome (PT: 10045170) | 6 | 30.10 (13.44-67.42) | 29.87 (166.12) | 4.89 (1.44) | 29.64 (13.23) |
| Hypophosphataemia (PT: 10021058) | 3 | 24.62 (7.89-76.80) | 24.53 (67.28) | 4.61 (0.38) | 24.38 (7.82) |
| **Respiratory, thoracic and mediastinal disorders (SOC: 10038738)** | | | | | |
| Tachypnoea (PT: 10043089) | 3 | 12.93 (4.15-40.27) | 12.89 (32.79) | 3.68 (0.25) | 12.85 (4.13) |
| Pneumonitis (PT: 10035742) | 3 | 6.67 (2.14-20.76) | 6.65 (14.38) | 2.73 (0.01) | 6.64 (2.13) |
| **Hepatobiliary disorders (SOC: 10019805)** | | | | | |
| Hypertransaminasaemia (PT: 10068237) | 4 | 19.91 (7.43-53.31) | 19.81 (71.07) | 4.30 (0.76) | 19.71 (7.36) |

In this stratified analysis, for both glofitamab and all other drugs, only reports of males were included. **Abbreviations:** PT, preferred term; ROR, reporting odds ratio; CI, confidence interval; PRR, proportional reporting ratio; χ2, chi-squared; IC, information component; IC025, lower limit of 95% confidence interval of IC; EBGM, empirical Bayesian geometric mean; EBGM05, lower limit of 95% confidence interval of EBGM.
