# Supplementary material for: Exploration of adverse event profiles for glofitamab: A disproportionality analysis using the FDA adverse event reporting system
Source: PLoS One. 2025 Nov 4;20(11):e0336151. doi: 10.1371/journal.pone.0336151 (PMC12585042; doi:10.1371/journal.pone.0336151)
Supplement: S10 Table — (DOCX) [file pone.0336151.s010.docx]

**S10 Table.** **Number and signal strength of glofitamab-related signals at the PT level stratified by females.**

| **PT** | **Number** | **ROR (95% CI)** | **PRR (χ2)** | **IC (IC025)** | **EBGM (EBGM05)** |
| --- | --- | --- | --- | --- | --- |
| **General disorders and administration site conditions (SOC: 10018065)** | | | | | |
| Death (PT: 10011906) | 30 | 6.73 (4.66-9.73) | 6.41 (137.97) | 2.68 (1.91) | 6.40 (4.43) |
| Pyrexia (PT: 10037660) | 24 | 9.19 (6.10-13.85) | 8.82 (167.05) | 3.14 (2.15) | 8.81 (5.85) |
| Hyperpyrexia (PT: 10020741) | 4 | 114.42 (42.53-307.85) | 113.56 (440.88) | 6.81 (0.96) | 112.19 (41.70) |
| **Immune system disorders (SOC: 10021428)** | | | | | |
| Cytokine release syndrome (PT: 10052015) | 48 | 223.87 (165.89-302.11) | 203.53 (9469.20) | 7.64 (4.87) | 199.16 (147.58) |
| Haemophagocytic lymphohistiocytosis (PT: 10071583) | 3 | 29.30 (9.40-91.30) | 29.14 (81.27) | 4.86 (0.41) | 29.05 (9.32) |
| **Investigations (SOC: 10022891)** | | | | | |
| Platelet count decreased (PT: 10035528) | 10 | 12.19 (6.52-22.81) | 11.98 (100.68) | 3.58 (1.70) | 11.97 (6.40) |
| Blood lactate dehydrogenase increased (PT: 10005630) | 4 | 54.44 (20.30-146.02) | 54.04 (207.04) | 5.75 (0.92) | 53.73 (20.03) |
| Alanine aminotransferase increased (PT: 10001551) | 4 | 11.15 (4.17-29.85) | 11.08 (36.65) | 3.47 (0.58) | 11.06 (4.13) |
| SARS-CoV-2 test positive (PT: 10084271) | 3 | 13.83 (4.44-43.06) | 13.76 (35.45) | 3.78 (0.26) | 13.74 (4.41) |
| Liver function test increased (PT: 10077692) | 3 | 10.47 (3.36-32.60) | 10.42 (25.53) | 3.38 (0.18) | 10.41 (3.34) |
| **Blood and lymphatic system disorders (SOC: 10005329)** | | | | | |
| Neutropenia (PT: 10029354) | 8 | 6.40 (3.18-12.87) | 6.32 (35.89) | 2.66 (1.02) | 6.32 (3.14) |
| Anaemia (PT: 10002034) | 7 | 5.60 (2.65-11.80) | 5.54 (26.06) | 2.47 (0.79) | 5.53 (2.62) |
| Thrombocytopenia (PT: 10043554) | 6 | 8.46 (3.78-18.93) | 8.38 (39.00) | 3.07 (0.93) | 8.37 (3.74) |
| **Nervous system disorders (SOC: 10029205)** | | | | | |
| Immune effector cell-associated neurotoxicity syndrome (PT: 10083347) | 5 | 59.02 (24.40-142.79) | 58.47 (280.71) | 5.86 (1.28) | 58.11 (24.02) |
| Neurotoxicity (PT: 10029350) | 3 | 28.20 (9.05-87.87) | 28.04 (78.02) | 4.81 (0.40) | 27.96 (8.97) |
| **Infections and infestations (SOC: 10021881)** | | | | | |
| Disseminated tuberculosis (PT: 10013453) | 3 | 206.21 (65.47-649.50) | 205.04 (595.90) | 7.65 (0.51) | 200.60 (63.69) |
| **Renal and urinary disorders (SOC: 10038359)** | | | | | |
| Haematuria (PT: 10018867) | 3 | 29.83 (9.57-92.97) | 29.67 (82.85) | 4.89 (0.41) | 29.58 (9.49) |
| **Respiratory, thoracic and mediastinal disorders (SOC: 10038738)** | | | | | |
| Respiratory failure (PT: 10038695) | 3 | 8.34 (2.68-25.96) | 8.30 (19.26) | 3.05 (0.10) | 8.29 (2.66) |

In this stratified analysis, for both glofitamab and all other drugs, only reports of females were included. **Abbreviations:** PT, preferred term; ROR, reporting odds ratio; CI, confidence interval; PRR, proportional reporting ratio; χ2, chi-squared; IC, information component; IC025, lower limit of 95% confidence interval of IC; EBGM, empirical Bayesian geometric mean; EBGM05, lower limit of 95% confidence interval of EBGM.
