# Supplementary material for: Exploration of adverse event profiles for glofitamab: A disproportionality analysis using the FDA adverse event reporting system
Source: PLoS One. 2025 Nov 4;20(11):e0336151. doi: 10.1371/journal.pone.0336151 (PMC12585042; doi:10.1371/journal.pone.0336151)
Supplement: S11 Table — (DOCX) [file pone.0336151.s011.docx]

**S11 Table.** **Number and signal strength of glofitamab-related signals at the PT level stratified by patients aged 18-65 years.**

| **PT** | **Number** | **ROR (95% CI)** | **PRR (χ2)** | **IC (IC025)** | **EBGM (EBGM05)** |
| --- | --- | --- | --- | --- | --- |
| **General disorders and administration site conditions (SOC: 10018065)** | | | | | |
| Pyrexia (PT: 10037660) | 36 | 11.89 (8.48-16.68) | 11.16 (334.35) | 3.48 (2.64) | 11.14 (7.94) |
| Death (PT: 10011906) | 28 | 8.70 (5.95-12.73) | 8.30 (180.60) | 3.05 (2.17) | 8.29 (5.66) |
| Hyperpyrexia (PT: 10020741) | 6 | 97.42 (43.31-219.13) | 96.34 (557.76) | 6.57 (1.61) | 94.92 (42.20) |
| **Immune system disorders (SOC: 10021428)** | | | | | |
| Cytokine release syndrome (PT: 10052015) | 48 | 138.98 (103.04-187.45) | 126.60 (5869.45) | 6.96 (4.71) | 124.16 (92.06) |
| **Investigations (SOC: 10022891)** | | | | | |
| Platelet count decreased (PT: 10035528) | 10 | 12.68 (6.78-23.72) | 12.46 (105.39) | 3.64 (1.73) | 12.44 (6.65) |
| Aspartate aminotransferase increased (PT: 10003481) | 4 | 10.70 (4.00-28.64) | 10.63 (34.86) | 3.41 (0.56) | 10.61 (3.97) |
| Alanine aminotransferase increased (PT: 10001551) | 4 | 8.40 (3.14-22.48) | 8.34 (25.85) | 3.06 (0.46) | 8.33 (3.11) |
| Blood lactate dehydrogenase increased (PT: 10005630) | 3 | 35.14 (11.26-109.63) | 34.94 (98.40) | 5.12 (0.43) | 34.76 (11.14) |
| **Blood and lymphatic system disorders (SOC: 10005329)** | | | | | |
| Neutropenia (PT: 10029354) | 9 | 5.38 (2.78-10.39) | 5.30 (31.49) | 2.41 (0.97) | 5.30 (2.74) |
| Anaemia (PT: 10002034) | 6 | 5.11 (2.29-11.43) | 5.07 (19.61) | 2.34 (0.58) | 5.06 (2.26) |
| Febrile neutropenia (PT: 10016288) | 4 | 6.48 (2.42-17.34) | 6.44 (18.38) | 2.69 (0.33) | 6.43 (2.40) |
| **Infections and infestations (SOC: 10021881)** | | | | | |
| Sepsis (PT: 10040047) | 6 | 8.02 (3.59-17.94) | 7.94 (36.41) | 2.99 (0.90) | 7.93 (3.55) |
| Septic shock (PT: 10040070) | 5 | 12.01 (4.97-29.00) | 11.91 (49.91) | 3.57 (0.89) | 11.89 (4.92) |
| **Respiratory, thoracic and mediastinal disorders (SOC: 10038738)** | | | | | |
| Pleural effusion (PT: 10035598) | 4 | 10.40 (3.89-27.83) | 10.33 (33.67) | 3.37 (0.55) | 10.31 (3.85) |
| Hypoxia (PT: 10021143) | 3 | 9.94 (3.19-30.95) | 9.89 (23.95) | 3.30 (0.17) | 9.88 (3.17) |
| Pneumonitis (PT: 10035742) | 3 | 12.53 (4.02-39.01) | 12.46 (31.59) | 3.64 (0.24) | 12.44 (4.00) |
| **Nervous system disorders (SOC: 10029205)** | | | | | |
| Movement disorder (PT: 10028035) | 5 | 18.69 (7.74-45.15) | 18.53 (82.70) | 4.21 (1.05) | 18.48 (7.65) |
| Immune effector cell-associated neurotoxicity syndrome (PT: 10083347) | 4 | 30.62 (11.42-82.08) | 30.40 (113.22) | 4.92 (0.84) | 30.26 (11.29) |

In this stratified analysis, for both glofitamab and all other drugs, only reports of patients aged 18-65 years were included. **Abbreviations:** PT, preferred term; ROR, reporting odds ratio; CI, confidence interval; PRR, proportional reporting ratio; χ2, chi-squared; IC, information component; IC025, lower limit of 95% confidence interval of IC; EBGM, empirical Bayesian geometric mean; EBGM05, lower limit of 95% confidence interval of EBGM.
