# Supplementary material for: Exploration of adverse event profiles for glofitamab: A disproportionality analysis using the FDA adverse event reporting system
Source: PLoS One. 2025 Nov 4;20(11):e0336151. doi: 10.1371/journal.pone.0336151 (PMC12585042; doi:10.1371/journal.pone.0336151)
Supplement: S12 Table — (DOCX) [file pone.0336151.s012.docx]

**S12 Table.** **Number and signal strength of glofitamab-related signals at the PT level stratified by patients aged >65 years.**

| **PT** | **Number** | **ROR (95% CI)** | **PRR (χ2)** | **IC (IC025)** | **EBGM (EBGM05)** |
| --- | --- | --- | --- | --- | --- |
| **Immune system disorders (SOC: 10021428)** | | | | | |
| Cytokine release syndrome (PT: 10052015) | 50 | 86.39 (64.37-115.93) | 78.25 (3738.64) | 6.26 (4.52) | 76.65 (57.11) |
| **General disorders and administration site conditions (SOC: 10018065)** | | | | | |
| Pyrexia (PT: 10037660) | 16 | 6.37 (3.87-10.47) | 6.20 (70.03) | 2.63 (1.53) | 6.19 (3.76) |
| Organ failure (PT: 10053159) | 4 | 146.96 (53.91-400.64) | 145.85 (553.52) | 7.13 (0.95) | 140.33 (51.47) |
| Hyperpyrexia (PT: 10020741) | 3 | 62.77 (19.99-197.13) | 62.42 (178.30) | 5.94 (0.47) | 61.39 (19.55) |
| Multiple organ dysfunction syndrome (PT: 10077361) | 3 | 7.01 (2.25-21.84) | 6.98 (15.35) | 2.80 (0.03) | 6.97 (2.24) |
| **Infections and infestations (SOC: 10021881)** | | | | | |
| Pneumonia (PT: 10035664) | 15 | 3.81 (2.28-6.38) | 3.73 (30.23) | 1.90 (0.94) | 3.73 (2.23) |
| Septic shock (PT: 10040070) | 7 | 12.04 (5.71-25.42) | 11.90 (69.71) | 3.57 (1.30) | 11.86 (5.62) |
| Disseminated tuberculosis (PT: 10013453) | 3 | 140.64 (44.26-446.89) | 139.85 (398.45) | 7.07 (0.49) | 134.77 (42.41) |
| **Nervous system disorders (SOC: 10029205)** | | | | | |
| Immune effector cell-associated neurotoxicity syndrome (PT: 10083347) | 7 | 32.04 (15.15-67.77) | 31.63 (205.95) | 4.97 (1.68) | 31.37 (14.83) |
| Neurotoxicity (PT: 10029350) | 4 | 20.75 (7.74-55.65) | 20.60 (74.22) | 4.36 (0.76) | 20.49 (7.64) |
| Cerebral haemorrhage (PT: 10008111) | 3 | 6.54 (2.10-20.36) | 6.51 (13.97) | 2.70 (0.00) | 6.50 (2.09) |
| **Investigations (SOC: 10022891)** | | | | | |
| Platelet count decreased (PT: 10035528) | 7 | 5.21 (2.47-10.98) | 5.15 (23.45) | 2.36 (0.73) | 5.15 (2.44) |
| Alanine aminotransferase increased (PT: 10001551) | 3 | 9.09 (2.92-28.33) | 9.05 (21.44) | 3.17 (0.13) | 9.03 (2.90) |
| **Renal and urinary disorders (SOC: 10038359)** | | | | | |
| Haematuria (PT: 10018867) | 3 | 8.92 (2.86-27.78) | 8.87 (20.92) | 3.15 (0.13) | 8.85 (2.84) |

In this stratified analysis, for both glofitamab and all other drugs, only reports of patients aged >65 years were included. **Abbreviations:** PT, preferred term; ROR, reporting odds ratio; CI, confidence interval; PRR, proportional reporting ratio; χ2, chi-squared; IC, information component; IC025, lower limit of 95% confidence interval of IC; EBGM, empirical Bayesian geometric mean; EBGM05, lower limit of 95% confidence interval of EBGM.
