# Supplementary material for: Exploration of adverse event profiles for glofitamab: A disproportionality analysis using the FDA adverse event reporting system
Source: PLoS One. 2025 Nov 4;20(11):e0336151. doi: 10.1371/journal.pone.0336151 (PMC12585042; doi:10.1371/journal.pone.0336151)
Supplement: S13 Table — (DOCX) [file pone.0336151.s013.docx]

**S13 Table.** **Number and signal strength of glofitamab-related signals at the PT level stratified by reports from Europe.**

| **PT** | **Number** | **ROR (95% CI)** | **PRR (χ2)** | **IC (IC025)** | **EBGM (EBGM05)** |
| --- | --- | --- | --- | --- | --- |
| **Immune system disorders (SOC: 10021428)** | | | | | |
| Cytokine release syndrome (PT: 10052015) | 84 | 117.18 (92.81-147.95) | 102.74 (8094.08) | 6.62 (5.18) | 98.18 (77.76) |
| Hypogammaglobulinaemia (PT: 10020983) | 4 | 11.88 (4.44-31.84) | 11.82 (39.42) | 3.56 (0.60) | 11.76 (4.39) |
| Haemophagocytic lymphohistiocytosis (PT: 10071583) | 3 | 8.56 (2.75-26.68) | 8.53 (19.88) | 3.09 (0.11) | 8.50 (2.73) |
| **Infections and infestations (SOC: 10021881)** | | | | | |
| COVID-19 (PT: 10084268) | 14 | 3.82 (2.25-6.48) | 3.76 (28.47) | 1.91 (0.91) | 3.75 (2.21) |
| Infection (PT: 10021789) | 12 | 6.52 (3.68-11.54) | 6.42 (54.89) | 2.68 (1.37) | 6.40 (3.61) |
| Septic shock (PT: 10040070) | 8 | 7.49 (3.73-15.06) | 7.42 (44.32) | 2.89 (1.14) | 7.39 (3.68) |
| Cytomegalovirus infection reactivation (PT: 10058666) | 3 | 8.00 (2.57-24.93) | 7.97 (18.23) | 2.99 (0.09) | 7.95 (2.55) |
| Sinusitis (PT: 10040753) | 3 | 9.21 (2.96-28.70) | 9.17 (21.77) | 3.19 (0.14) | 9.14 (2.93) |
| COVID-19 pneumonia (PT: 10084380) | 3 | 7.54 (2.42-23.47) | 7.51 (16.87) | 2.90 (0.06) | 7.48 (2.40) |
| Upper respiratory tract infection (PT: 10046306) | 3 | 8.55 (2.75-26.64) | 8.52 (19.84) | 3.09 (0.11) | 8.49 (2.73) |
| Disseminated tuberculosis (PT: 10013453) | 3 | 40.72 (12.96-127.90) | 40.54 (113.62) | 5.32 (0.43) | 39.83 (12.68) |
| **General disorders and administration site conditions (SOC: 10018065)** | | | | | |
| Death (PT: 10011906) | 27 | 3.66 (2.49-5.38) | 3.55 (50.02) | 1.83 (1.14) | 3.55 (2.41) |
| Pyrexia (PT: 10037660) | 18 | 4.03 (2.52-6.45) | 3.95 (39.91) | 1.98 (1.10) | 3.95 (2.47) |
| **Blood and lymphatic system disorders (SOC: 10005329)** | | | | | |
| Neutropenia (PT: 10029354) | 17 | 3.74 (2.31-6.06) | 3.67 (33.21) | 1.87 (0.98) | 3.67 (2.26) |
| **Nervous system disorders (SOC: 10029205)** | | | | | |
| Immune effector cell-associated neurotoxicity syndrome (PT: 10083347) | 16 | 55.86 (33.82-92.28) | 54.56 (821.23) | 5.74 (2.99) | 53.26 (32.24) |
| **Metabolism and nutrition disorders (SOC: 10027433)** | | | | | |
| Tumour lysis syndrome (PT: 10045170) | 4 | 14.80 (5.52-39.68) | 14.72 (50.83) | 3.87 (0.67) | 14.63 (5.46) |
| Hypophosphataemia (PT: 10021058) | 3 | 16.25 (5.20-50.71) | 16.18 (42.42) | 4.01 (0.30) | 16.07 (5.15) |
| **Investigations (SOC: 10022891)** | | | | | |
| Blood lactate dehydrogenase increased (PT: 10005630) | 3 | 8.84 (2.84-27.54) | 8.80 (20.68) | 3.13 (0.12) | 8.77 (2.82) |
| SARS-CoV-2 test positive (PT: 10084271) | 3 | 20.36 (6.52-63.61) | 20.27 (54.47) | 4.33 (0.34) | 20.10 (6.43) |
| **Hepatobiliary disorders (SOC: 10019805)** | | | | | |
| Hypertransaminasaemia (PT: 10068237) | 3 | 7.48 (2.40-23.28) | 7.45 (16.70) | 2.89 (0.06) | 7.43 (2.38) |

In this stratified analysis, for both glofitamab and all other drugs, only reports from Europe were included. **Abbreviations:** PT, preferred term; ROR, reporting odds ratio; CI, confidence interval; PRR, proportional reporting ratio; χ2, chi-squared; IC, information component; IC025, lower limit of 95% confidence interval of IC; EBGM, empirical Bayesian geometric mean; EBGM05, lower limit of 95% confidence interval of EBGM.
