# Supplementary material for: Exploration of adverse event profiles for glofitamab: A disproportionality analysis using the FDA adverse event reporting system
Source: PLoS One. 2025 Nov 4;20(11):e0336151. doi: 10.1371/journal.pone.0336151 (PMC12585042; doi:10.1371/journal.pone.0336151)
Supplement: S14 Table — (DOCX) [file pone.0336151.s014.docx]

**S14 Table.** **Number and signal strength of glofitamab-related signals at the PT level stratified by reports from Asia.**

| **PT** | **Number** | **ROR (95% CI)** | **PRR (χ2)** | **IC (IC025)** | **EBGM (EBGM05)** |
| --- | --- | --- | --- | --- | --- |
| **General disorders and administration site conditions (SOC: 10018065)** | | | | | |
| Death (PT: 10011906) | 42 | 3.74 (2.73-5.13) | 3.53 (77.73) | 1.82 (1.28) | 3.53 (2.57) |
| Pyrexia (PT: 10037660) | 38 | 5.70 (4.10-7.93) | 5.38 (136.59) | 2.42 (1.79) | 5.36 (3.85) |
| Hyperpyrexia (PT: 10020741) | 8 | 21.96 (10.85-44.41) | 21.65 (154.81) | 4.41 (1.73) | 21.27 (10.52) |
| Organ failure (PT: 10053159) | 4 | 75.80 (27.48-209.07) | 75.26 (275.34) | 6.14 (0.90) | 70.76 (25.65) |
| Temperature intolerance (PT: 10057040) | 3 | 8.60 (2.75-26.85) | 8.56 (19.89) | 3.09 (0.11) | 8.50 (2.72) |
| **Immune system disorders (SOC: 10021428)** | | | | | |
| Cytokine release syndrome (PT: 10052015) | 28 | 22.76 (15.51-33.41) | 21.66 (542.97) | 4.41 (3.09) | 21.28 (14.50) |
| **Infections and infestations (SOC: 10021881)** | | | | | |
| COVID-19 (PT: 10084268) | 10 | 3.89 (2.08-7.28) | 3.84 (21.02) | 1.94 (0.72) | 3.83 (2.05) |
| Infection (PT: 10021789) | 10 | 5.87 (3.13-10.98) | 5.78 (39.43) | 2.52 (1.12) | 5.75 (3.07) |
| Herpes zoster (PT: 10019974) | 5 | 7.91 (3.27-19.14) | 7.85 (29.72) | 2.96 (0.68) | 7.80 (3.23) |
| **Investigations (SOC: 10022891)** | | | | | |
| Platelet count decreased (PT: 10035528) | 16 | 3.65 (2.22-6.00) | 3.57 (29.76) | 1.83 (0.92) | 3.56 (2.17) |
| Blood bilirubin increased (PT: 10005364) | 5 | 8.36 (3.46-20.24) | 8.30 (31.89) | 3.04 (0.71) | 8.24 (3.41) |
| **Nervous system disorders (SOC: 10029205)** | | | | | |
| Movement disorder (PT: 10028035) | 7 | 30.50 (14.34-64.90) | 30.13 (192.26) | 4.88 (1.65) | 29.40 (13.82) |
| **Metabolism and nutrition disorders (SOC: 10027433)** | | | | | |
| Tumour lysis syndrome (PT: 10045170) | 3 | 7.76 (2.49-24.23) | 7.72 (17.46) | 2.94 (0.07) | 7.68 (2.46) |
| Feeding disorder (PT: 10061148) | 3 | 7.56 (2.42-23.60) | 7.53 (16.88) | 2.90 (0.06) | 7.48 (2.40) |

In this stratified analysis, for both glofitamab and all other drugs, only reports from Asia were included. **Abbreviations:** PT, preferred term; ROR, reporting odds ratio; CI, confidence interval; PRR, proportional reporting ratio; χ2, chi-squared; IC, information component; IC025, lower limit of 95% confidence interval of IC; EBGM, empirical Bayesian geometric mean; EBGM05, lower limit of 95% confidence interval of EBGM.
