# Supplementary material for: Exploration of adverse event profiles for glofitamab: A disproportionality analysis using the FDA adverse event reporting system
Source: PLoS One. 2025 Nov 4;20(11):e0336151. doi: 10.1371/journal.pone.0336151 (PMC12585042; doi:10.1371/journal.pone.0336151)
Supplement: S15 Table — (DOCX) [file pone.0336151.s015.docx]

**S15 Table.** **Number and signal strength of glofitamab-related signals at the PT level stratified by** **reports from America.**

| **PT** | **Number** | **ROR (95% CI)** | **PRR (χ2)** | **IC (IC025)** | **EBGM (EBGM05)** |
| --- | --- | --- | --- | --- | --- |
| **Immune system disorders (SOC: 10021428)** | | | | | |
| Cytokine release syndrome (PT: 10052015) | 34 | 356.41 (247.34-513.58) | 304.55 (10186.55) | 8.24 (4.45) | 301.45 (209.20) |
| **Nervous system disorders (SOC: 10029205)** | | | | | |
| Immune effector cell-associated neurotoxicity syndrome (PT: 10083347) | 6 | 118.51 (52.60-267.00) | 115.49 (678.46) | 6.85 (1.62) | 115.04 (51.06) |
| Neurotoxicity (PT: 10029350) | 5 | 124.40 (51.19-302.28) | 121.75 (596.43) | 6.92 (1.33) | 121.25 (49.90) |
| **Investigations (SOC: 10022891)** | | | | | |
| Alanine aminotransferase increased (PT: 10001551) | 3 | 30.95 (9.90-96.74) | 30.57 (85.75) | 4.93 (0.41) | 30.54 (9.77) |
| Aspartate aminotransferase increased (PT: 10003481) | 3 | 37.14 (11.88-116.09) | 36.68 (104.02) | 5.20 (0.43) | 36.63 (11.72) |
| **Blood and lymphatic system disorders (SOC: 10005329)** | | | | | |
| Neutropenia (PT: 10029354) | 5 | 13.71 (5.65-33.27) | 13.44 (57.64) | 3.75 (0.93) | 13.43 (5.54) |
| **General disorders and administration site conditions (SOC: 10018065)** | | | | | |
| Disease recurrence (PT: 10061819) | 3 | 8.67 (2.77-27.08) | 8.57 (20.08) | 3.10 (0.11) | 8.57 (2.74) |

In this stratified analysis, for both glofitamab and all other drugs, only reports from America were included. **Abbreviations:** PT, preferred term; ROR, reporting odds ratio; CI, confidence interval; PRR, proportional reporting ratio; χ2, chi-squared; IC, information component; IC025, lower limit of 95% confidence interval of IC; EBGM, empirical Bayesian geometric mean; EBGM05, lower limit of 95% confidence interval of EBGM.
